# Experimental evidence of pollination by deception in a dioecious palm

**DOI:** 10.1186/s12862-025-02388-6

**Published:** 2025-05-12

**Authors:** Galilea Orellana-Vera, Thomas Auffray, Rommel Montúfar, Marc Gibernau, Sylvain Pincebourde, Arturo Guasti, Jérôme Casas, Olivier Dangles

**Affiliations:** 1 PUCE, Facultad de Ciencias Exactas y Naturales, Quito, Ecuador; 2 CEFE, Université de Montpellier, CNRS, EPHE, Montpellier, IRD France; 3 LSPE, CNRS, Université de Corse, Ajaccio, France; 4 IRBI, CNRS, Université de Tours, Tours, France; 5 OTONGA Foundation, Quito, Ecuador

**Keywords:** Daily variation, Deceptive pollination, Entomophily, Ivory palm, Sexual dimorphism

## Abstract

**Background:**

Flower traits and pollinator activity patterns can vary over the course of a single day. Therefore, the pollination processes occurring over short time scales are crucial to sustain the complex dynamics of plant-pollinator interactions. Here, we characterized the diel patterns of flower opening (*e.g.* anthesis), scent emission, and insect visits in highly dimorphic male (rewarding) and female (deceptive) inflorescences of the ivory palm (*Phytelephas aequatorialis)*, a thermogenic dioecious species endemic to western Ecuador. We conducted field experiments using artificial scented-baits (designated as artificial flowers) consisting of a heating plate (simulating thermogenesis) and p-methylanisole (the primary odor compound in inflorescences of both sexes) in two different amounts to mimic female and male inflorescences.

**Results:**

We found that female inflorescences open synchronously at dawn and dusk, while male inflorescences can open at any time throughout the day. Both sexes emitted floral odors consistently throughout the day. Even though male inflorescences emitted greater quantities of p-methylanisole, artificial flowers with different amounts of p-methylanisole attracted a similar diversity and abundance of insects throughout the day. Furthermore, male and female artificial flower attracted an equal abundance of visitors within five minutes of the emission of p-methylanisole.

**Conclusions:**

The findings suggest that, despite sexual dimorphism in opening time, intersexual mimicry in *P. aequatorialis* is sustained by a consistent odor release, which optimizes the probability of both sexes being visited by the same insect community during the day.

**Supplementary Information:**

The online version contains supplementary material available at 10.1186/s12862-025-02388-6.

## Introduction

Pollination success depends on the temporal synchrony between partners, which can vary significantly over time intervals as large as years or as precise as minutes or seconds [1]. Studies that deal with plant-pollinator communities over long periods may be biased, as fluctuations in pollinator composition and abundance on a 24-h scale can affect the timing of encounters with host plants, and hence their reproductive success [2]. Daily temporal resolution provides fine-scale dynamics of pollination-related processes that vary according to numerous factors, including environmental factors (temperature, solar radiation, wind speed, and lunar cycle) and biotic factors (*e.g.*, predation, competition, and physiology including the circadian clock) [1]. Experiments in pollination networks have demonstrated the importance of a short temporal resolution [3] when quantifying processes such as flower opening, the release of attractive floral scents, and the general availability of floral rewards that all can exhibit daily rhythms [4]. For instance, the"push–pull interaction"strategy is a such diel pattern of emission, in which the flowers attract then repel the pollinators by modulating the composition [5] and/or quantity [6] of volatile emission of their flowers over the course of a day. Similarly, pollinators have a daily rhythm of activity and receptivity to stimuli, so their abundance and richness around flowers can vary more within a day than between days [7]. To increase synchrony levels, many plants have evolved the ability to intensify odor release precisely at the peak of insect activity [8].

Synchrony between plants and pollinators is especially crucial in the case of dioecious plants (at least 15,600 species worldwide) in which male and female unisexual flowers are spatially separated in distinct individuals [9]. The separation of the sexes suggests that they may have responded differently to the same evolutionary pressures, leading to asymmetrical investments in sexual expression and different strategies to reach their fitness optima, sometimes in conflict with pollinator preferences [10]. Moreover, the sexes can compete with each other to attract pollinating insects separately [11]. Sexual selection shapes traits that influence mating success, primarily through intrasexual competition for access to mates [12]. In contrast, pollinator-mediated selection acts on floral traits favored by pollinators, driving adaptations that enhance pollination efficiency [13]. Both evolutionary pressures in male flowers act on opening phenology and enhance the investment in floral traits to increase their attractiveness to pollinators, such as bright colors, seductive scents, specific shapes and sizes, and reward production [14]. Male flowers are less constrained than females in allocating resources to pollinator-attractive traits [10]. Additionally, male plants compete with each other for pollinators [10]. Conversely, female plants should allocate resources to the production of offspring, thereby facing a trade-off between attracting pollinators and avoiding seed predators [10]. Consequently, female plants tend to display more subtle floral traits than those displayed by males [13].

A common pollination mechanism in dioecious models is deception, especially when only one sex offers a reward [15]. The deceptive sex must maintain signals that mimic the rewarding sex to attract pollinators and maximize fitness [16]. To explain the maintenance of daily mutualistic interactions with pollinators in dioecious and deceptive systems, the “no preference” hypothesis suggests that pollinators cannot distinguish between male and female flowers because there are no detectable differences between them [17], including no differences in the timing of signal display. Unfortunately, this “no preference” hypothesis has only rarely been tested in dioecious/deceptive plants with a temporal resolution high enough, at the minute/hourly time step, to detect any subtle mismatch.

We tested the “no preference” hypothesis in the dioecious palm *Phytelephas aequatorialis* (Arecaceae). This palm shows intersexual chemical mimicry through the emission of a floral odor composed almost entirely of the volatile compound p-methylanisole in both sexes (up to 99% of the total chemical profile), which is a key attractant for specific inflorescence-visiting insects [18] (Auffray T, Beugnon R, Orellana G, Guasti A, Pincebourde S, Montúfar R, Dangles O, Gibernau M: Hot cheating: intersexual mimicry underlies efficient pollinator attraction in a dioecious thermogenic palm, unpublished). In a recent study, we found that male inflorescences emit two to three times more p-methylanisole than females within 24 h after the anthesis started (Auffray T, Beugnon R, Orellana G, Guasti A, Pincebourde S, Montúfar R, Dangles O, Gibernau M: Hot cheating: intersexual mimicry underlies efficient pollinator attraction in a dioecious thermogenic palm, unpublished). However, females emit the p-methylanisole during more than four days, when males emit during up to only two days. During the opening days, the inflorescences are visited by a diverse community of insects at different periods of the day according to their windows of activity (Auffray T, Beugnon R, Orellana G, Guasti A, Pincebourde S, Montúfar R, Dangles O, Gibernau M: Hot cheating: intersexual mimicry underlies efficient pollinator attraction in a dioecious thermogenic palm, unpublished). The insect community of inflorescence visitors of *P. aequatorialis* comprises approximately sixty species, largely dominated by Coleoptera (cantharophily) particularly belonging to the families Staphylinidae, Curculionidae, and Nitidulidae [19] (Auffray T, Beugnon R, Orellana G, Guasti A, Pincebourde S, Montúfar R, Dangles O, Gibernau M: Hot cheating: intersexual mimicry underlies efficient pollinator attraction in a dioecious thermogenic palm, unpublished). The male inflorescences have developed a mutualistic relationship with a diverse community of visitors that transport pollen [20]. These visitors benefit from resources such as nourishment for adults and larvae, sites for reproduction, oviposition and predation of other visitors, shelter from predators, and a favorable microclimate within the inflorescence [20]. However, the female inflorescences deceive their visitors by mimicking the male olfactory signal, offering apparently no reward [12] (Auffray T, Beugnon R, Orellana G, Guasti A, Pincebourde S, Montúfar R, Dangles O, Gibernau M: Hot cheating: intersexual mimicry underlies efficient pollinator attraction in a dioecious thermogenic palm, unpublished).

We designed an artificial flower (AF) to standardize the methodology used for assessing chemical attraction in dioecious models of deception with odor being the only component of variation. The use of artificial flowers minimizes external factors in real inflorescences that could affect the results, such as the potential bias from the assumption that insects have learned the location of inflorescences and intrasexual variations in inflorescence size and volatile organic compound (VOC) emissions [13]. The deployment of an insect-trapping mechanism mimicking a true flower enabled us to select a specific location and moment of the day for the experiment, within a designated time window, and to monitor visitors at a high frequency. The details of the AF can be accessed in the Materials and Methods section and Online Resource 1. Remarkably, p-methylanisole exhibits specificity in mediating interactions with inflorescence-visiting insects and is also available as a commercial product. This allowed us to conduct field experiments in which we could manipulate the amount of floral odor in natural populations of inflorescence visitors, mimicking the specificity of both male and female inflorescences of *P. aequatorialis*.

Through experiments with the AFs, we aimed to understand the daily dynamics of both insects and inflorescences under field-controlled conditions. The primary hypothesis is that visitors of *P. aequatorialis* are unable to discriminate between male and female inflorescences on the basis of their p-methylanisole emission, suggesting chemical intersexual mimicry. Consequently, two predictions emerge. First, (1a) from the perspective of the insects, male and female inflorescences will be synchronized on diel patterns of anthesis and pattern of volatile emissions throughout the day. Second, (1b) from the perspective of the inflorescences, at the end of the inflorescences'functional lifespan, the composition and abundance of the visiting insect community will be similar throughout the day across two p-methylanisole quantity treatments in AFs that resemble real male and female inflorescences. Alternatively, if insects can differentiate between male and female inflorescences, it is predicted that (2a) each inflorescence will exhibit a unique pattern of opening and odor emission within a day. Furthermore, (2b) insects would be found at a remarkably lower abundance at the end of the functional phase of the unrewarding female inflorescence represented by a treatment with a lower quantity of p-methylanisole in the AF.

## Materials and methods

### Biological system

**Fig. 1:**
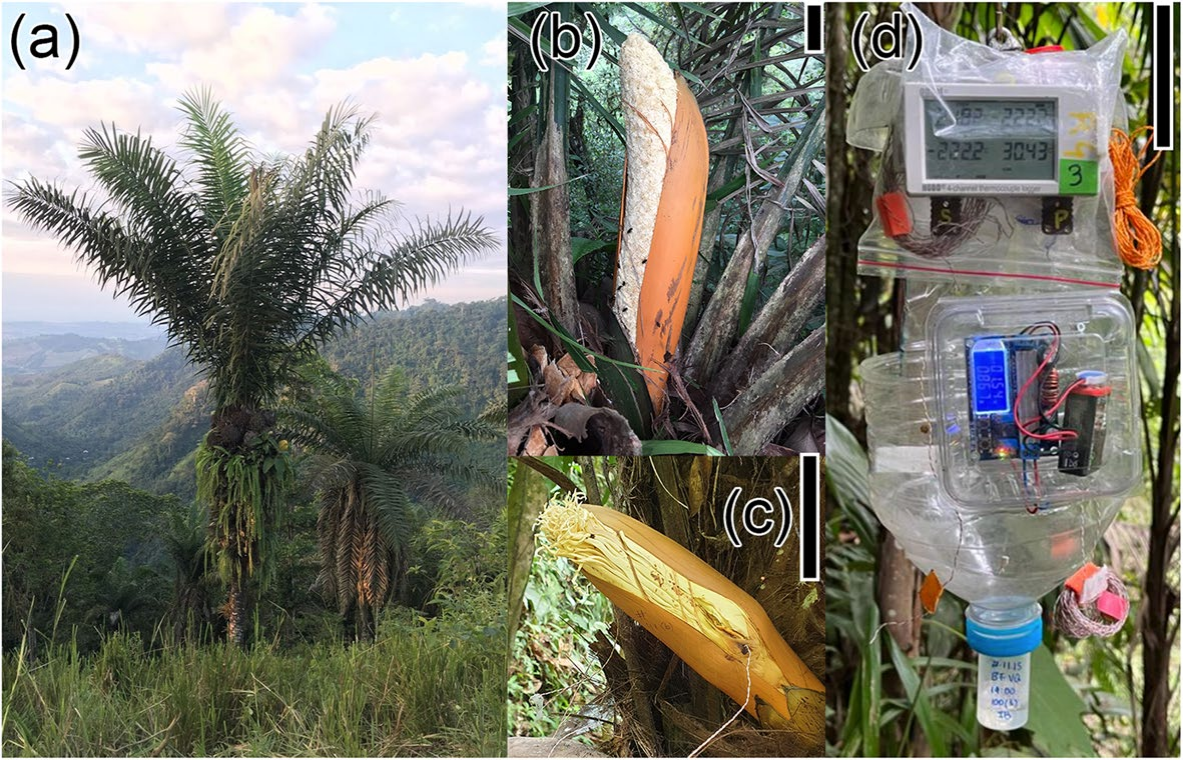
The dioecious tropical palm *Phytelephas aequatorialis*. **a** A female individual, (**b**) male, and (**c**) female inflorescences early opening by bract cracking. **d** Artificial flower. Scale bars represent 10 cm

*Phytelephas aequatorialis* Spruce (Arecaceae), is a dioecious sexually dimorphic endemic palm of Ecuador (locally known as tagua) found in tropical environments of western Ecuador and in subtropical regions of the Andean foothills [21]. *P. aequatorialis* is a keystone species because of its ecological role in providing habitat and food for several organisms [22]. It is a multipurpose palm, known mainly for its solid, whitish ivory-like endosperm (tagua) and its leaves used in roof construction [22]. Normally one inflorescence is in anthesis at a time per individual, although we observed sometimes that individuals from both sexes exhibit several functional inflorescences at once [22]. At the bud stage, both inflorescences are enclosed within modified yellowish leaves, designated as bracts (Fig. 1B, C). The male inflorescence is a pendant spike containing hundreds of flowers (1–2 cm) with pollen-filled stamens (1–2 cm long) on a rachis up to two meters long [23]. In contrast, the female inflorescence has 10–20 flowers up to 40 cm long [23].

### Study site

The study was conducted in the Otongachi Ecological Reserve, situated in the western Andean foothills of Ecuador and extending from 850 to 1,150 m above sea level (0°19'S, 78°57'W). The reserve is located at the periphery of the Pichincha province, before the city of “La Unión del Toachi”. The Otongachi Ecological Reserve is approximately 150 hectares in size and is composed of primary forest, secondary forest, grassland in smaller proportions and a botanical garden with useful plant species [24]. The forest belongs to the Chocó ecoregion and consists of an evergreen secondary foothill forest with tree and shrub species, bamboo, epiphytes, mosses, creepers and palms [24]. The mean temperature and relative humidity at the study site were 20.5 °C ± 1.37 °C and 92.28% ± 6.65%, respectively, as reported by external temperature sensor Data Logger (HOBO MX2304, Bourne, USA) that captured data every minute during the field periods in 2022 and 2023. The flowering season of *P. aequatorialis* occurs during the dry season, from August to February. We conducted the study in a 10-ha plot containing a wild population of *P. aequatorialis* (Fig. 1A), situated at an elevation of 850 m above sea level.

### Time of bract opening and flowering stage monitoring

We monitored the flowering of 29 inflorescences of *P. aequatorialis* (15 females and 14 males) from 20-Jul-2021 to 24-Sep-2021, and from 9-Aug-2022 to 14-Oct-2022. We placed time-lapse cameras (TLC200 Pro, Brinno, Taipei, Taiwan, with additional light at night or model 119,576, Bushnell Trophy Cam HD, China, infrared trail camera), with time-lapse mode set at 1 min intervals, in front of closed bracts to record the onset of flowering. Based on detailed observations of the time-lapse videos, we defined three main phases in the sequence of the inflorescence opening."Bract cracking"refers to the appearance of a vertical crack in the bract."Deployment start"indicates the beginning of deployment or decompression of the floral tissues outside the bract. We considered the inflorescence to be"fully deployed'when we no longer observed any sign of extension. For photographic references to these phases in the inflorescences, see Online Resource 4.

### Daily emission of floral scent

#### Odour sampling

We studied the diurnal variation of volatile organic compound (VOC) emissions from a different set of 27 inflorescences of *P. aequatorialis* (12 females and 15 males) from 20-Jul-2021 to 25-Sep-2021, and from 8-Aug-2022 to 24-Nov-2022. Twenty-one inflorescences are included among the 29 in which we observed floral opening (as reported in the previous section) and the 27 from which we collected odors (as reported in this section). This means that in 21 inflorescences we collected both types of data. We used the headspace technique coupled to a chromatoprobe, a glass cartridge filled with 1.5 mg of Carbotrap (20–40 mesh) and 1.5 mg of Tenax® TA (60–80 mesh, Supelco) sealed at both ends with glass wool [25, 26]. Prior to analysis, each chromatoprobe was conditioned in the laboratory for 1 h at 250 °C, with an N_2_ flow from 20 to 50 mL min^−1^. A solution of 1 µL of dodecane (100 µg/µL) mixed with dichloromethane was previously injected into each chromatoprobe to allow accurate quantification of the collected VOCs. In the field, we completely enclosed a female inflorescence with an odorless polyethylene terephthalate bag (Nalophane®, 50 × 50 cm) tied at both ends with cotton strings. By contrast, it was not possible to completely cover the male inflorescences when they reached their maximum size at anthesis (≥ 1 m). We therefore covered a portion of the male inflorescence (typically 1/2 or ¾ of the length) and estimated the VOC emission of the entire inflorescence. This was achieved by multiplying the total length of the inflorescence by 100 and dividing by the surface of the portion measured. Once in the bag, we connected the chromatoprobe to a Tygon® tube connected to a pump unit (KNF NMP830 KPDC, KNF Neuberger SAS, France), with flow rate finely regulated using flowmeters (ZLVR-VAF-1012819–1, Swagelok Lyon, France). The pump was set to aspirate the air from the bag for 15 min at a flow rate of 200 mL min^−1^. After collection, the chromatoprobes were preserved in a glass vial and stored in the refrigerator (−20 °C) until the analysis. We sampled each inflorescence three times a day (one sample in each of the following daily intervals 6:00—10:00, 12:00—15:00, and 17:30—21:00) for three days from the first day of anthesis (since bract cracking) for females and the first two days for males. In the end, we recorded 59.25 h of VOC sampling (138 female samples for a total of 34.5 h and 99 male samples for 24.75 h). The presence of airborne contaminants was checked by collecting one to four blank samples per inflorescence by sampling the air from an empty Nalophan bag previously opened a few meters from the inflorescences [27].

#### VOC analysis

Chemical analyses were performed at the Platform for Chemical Analyses in Ecology (PACE, Montpellier, France). We analyzed the VOCs collected on chromatoprobes using a gas chromatograph (GC, Trace 1310, Thermo ScientificTM, Milan, Italy) equipped with an Optima 5-MS capillary column (30 m length, 0.25 mm internal diameter, 0.25 µm film thickness; Macherey–Nagel, Düren, Germany) coupled to a mass spectrometer (ISQ QD Single Quadrupole, Thermo ScientificTM, Milan, Italy) and helium as carrier gas at a rate of 1 mL min^−1^. Chromatoprobes were desorbed individually in a thermal desorption unit (TDU, Gerstell, Mülheim, Germany) at 250 °C in spitless mode. VOCs were injected at a 1:4 split ratio into a cold injection system (CIS-multi-purpose sampler, Gerstell, Mülheim, Germany), where they were condensed with liquid nitrogen at −80 °C and then heated to 250 °C for transfer to the gas chromatograph. The oven parameters were set as follows: held at 40 °C for 3 min, increased from 40 °C to 220 °C at a rate of 5 °C min^−1^ and from 220 to 250 °C at 10 °C min^−1^, and finally held at 250 °C for 2 min. The acquisition parameters of the mass spectrometer were set to detect mass ranges from 38 m z^−1^ (mass-to-charge ratio) to 350 m z^−1^ at an ionization energy of 70 eV.

The data were processed using MZmine3 (version 3.4) [28]. The identity of the detected VOCs was confirmed using the retention index method and the spectral comparison with both the NIST17 database and the spectra library available in the laboratory. Retention indices were determined by injecting a series of n-alkanes (Alkanes Standard Solution, Sigma Aldrich, Munich, Germany) several times between the chromatoprobe analyses. Contaminants (column leaks, phthalates) and ambient air contaminants were discarded from the final list of VOCs. The emission of each VOC was quantified by comparing the peak area of each VOC with the area of the dodecane in each chromatogram. To gain a deeper understanding of the daily perspective of a visiting insect attracted by floral traits, we integrated both the time of the day and the age of the inflorescence at sampling in the analyses. However, we are aware that if we consider the passage of time from anthesis, there may be a different VOC pattern (Auffray T, Beugnon R, Orellana G, Guasti A, Pincebourde S, Montúfar R, Dangles O, Gibernau M: Hot cheating: intersexual mimicry underlies efficient pollinator attraction in a dioecious thermogenic palm, unpublished).

### Field experiments with artificial flowers

We designed an artificial flower (hereafter referred to as AF, Fig. 1D) by placing a petri dish inside an interception trap, which diffuses a certain amount of commercial p-methylanisole over a Peltier module to simulate thermogenesis (for more details see the Online Resource 1). We programmed the system such that the temperature of the Peltier module was always 10 °C above the ambient temperature for both the male- and female-mimicking setups. The maximal temperatures reached by real thermogenic inflorescences do not differ between the two sexes (Auffray T, Beugnon R, Orellana G, Guasti A, Pincebourde S, Montúfar R, Dangles O, Gibernau M: Hot cheating: intersexual mimicry underlies efficient pollinator attraction in a dioecious thermogenic palm, unpublished). We built ten identical units of this AF.

In Experiment #1 Daily Mimicry, we assessed the influence of the quantity of p-methylanisole emitted by male and female inflorescences on the diversity and abundance of visiting insects as function of the time of day. We placed five AFs at 15 m intervals along a transect on a forest track in the Otongachi Ecological Reserve (0°19′15.721"S; 78°57′5.488"W) in an area sparsely populated with *P. aequatorialis* trees to avoid potential interference with real inflorescences at anthesis (we did not observe inflorescences nearby during the experiment). In two AFs (randomly selected) we applied 50 μL of p-methylanisole (named female AF) to the diffuser to mimic p-methylanisole emission from female inflorescences. In two other AFs chosen randomly, we added 400 μL of p-methylanisole (named male AF) to mimic odor release from male inflorescences, which, at their maximum yield, release approximately eight times more odor than that released by female inflorescences (see Online Resource 1 for more details on the choice of the p-methylanisole amounts to mimic female and male inflorescences). The last AF was a control in which we did not add p-methylanisole to ensure that only the VOC deposited in an AF is attractive to insects. We ran the experiment between 06:00 and 21:00, changing the insect collector, battery, and p-methylanisole every hour. We repeated the experiment for five non-consecutive days, and an additional night sampling (from 16:00 to 21:00), from 24-Nov-2023 to 17-Dec-2023, randomizing the assignment (male or female AF or control) of the traps each day. We conducted 10 time series with artificial flowers, including five time series with a control AF. In total, male and female AFs operated for 156 h (corresponding to five days of sampling and two replicates of each treatment per sampling), and the control AF for 78 h. We stored the insects in tubes with 90% alcohol, identified morphospecies until family taxonomic level (based on previous assignments) [19], and counted them using an OLYMPUS SZ61 (SZ2-ILST) stereomicroscope. Reference specimens were conserved in the collection of the Museum of Zoology QCAZ (QCAZ-002-JIP-2023) of the Pontificia Universidad Católica del Ecuador (PUCE). Since few taxa dominated the community of visiting insects (mean Simpson index = 0.95 and 0.96 for female and male AF, respectively), we focused the subsequent analyses on 16 morphospecies that represented 90% of the total abundance [19].

In Experiment #2 Early Attraction, we determined the arrival sequence of insect morphospecies at the two p-methylanisole concentrations using the same field design as above (two female AF, two male AF, and one control) in another transect of the Otongachi Ecological Reserve (0°19′13.789"'S; 78°57′6.036"W) from 12-Dec-2023 to 19-Dec-2023. The sampling lasted for 20 min, which is sufficient time to capture a majority of the 16 morphospecies most abundant found on true inflorescences, out of a total of 34 morphospecies captured at the end of all 20-min repetitions. During this period, we removed and replaced the insect collectors with new ones every five minutes. We performed three replicates within each period of highest insect activity, i.e., consecutive hours in the dawn from 06:00 to 08:00 and in the dusk from 17:00 to 19:00 [19], spaced by 40 min, time considered sufficient for the p-methylanisole odorant plume to fade between experiments, thus avoiding interference from insects attracted in the previous trial. We repeated the experiment on three non-consecutive days (dawn/dusk), and an additional dusk sampling, randomizing the trap assignment. In the end, we obtained six time series in the dawn and dusk for male and female AFs, representing a total of 800 min equivalent to 13.33 h of sampling for male AF and for female AF and 400 min (6.67 h) for the control. Insects were processed, counted, and identified as explained above.

### Statistical analyses

We performed all statistical analyses with the R statistical software (version 4.4.0) [29] and the R Studio interface (version 1.4.1717) [30].

#### Flowering phenology

We used a non-parametric kernel density estimation technique to determine the diel pattern of opening events in female and male inflorescences, utilizing the “overlap” package [31]. We then proceeded as described by [32], by plotting fitted circular kernel distribution curves and overlapping curves for both sexes. We calculated the overlap coefficient using the Dhat1 estimator, performed a smoothed bootstrap (10,000 resamples) for the overlap index, and calculated the confidence interval using basic0 and a logistic scale.

#### Emission rate of p-methylanisole

Since maximum performance is a suitable index for the analysis of physiological activity [33], and to avoid any bias attributed to low values resulting from errors in odor detection, chemical analysis, inflorescence age, and time of collection [34], we filtered 75% of the maximum values of p-methylanisole release for the generalized additive model (GAM). We used the gam function of the “mgcv” R package defined under the family distribution gamma (link = “log”), and a restricted maximum likelihood (RELM) [35]. The model considers odor emission as a function of time (minutes and days from anthesis), inflorescence sex, and the identity of the sampled inflorescences. Details are provided in the Online Resource 2. Using the R package “MuMIn”*,* we selected the best model by comparing AIC values (Akaike Information Criterion) [36] and checked the quality by confirming the residuals were normally distributed and homoscedastic.

#### Insect communities

For the Experiment #1 Daily Mimicry, we used the “vegan” package to calculate alpha diversity indices for each hour [37] and performed a Kruskal–Wallis test to compare the distribution of insect abundance attracted to female and male AF over the hours of the day. We used the “dunn.test” package for the following post-hoc test using the Bonferroni correction when necessary [38]. Additionally, we performed a Generalized Linear Mixed Model (GLMM) using the package “lme4”, which takes into account the number of insects captured as a function of the amount of p-methylanisole and the time of day, and as a random factor the time series [39]. In Experiment #2 Early Attraction, we performed a non-parametric Quade test to compare the mean number of insects attracted in five-minute time intervals. The blocking factor, necessary for this analysis, was the p-methylanisole quantity (50 μL for female AF and 400 μL for male AF) in both periods of the day (dawn and dusk).

## Results

### Daily flower opening

**Fig. 2:**
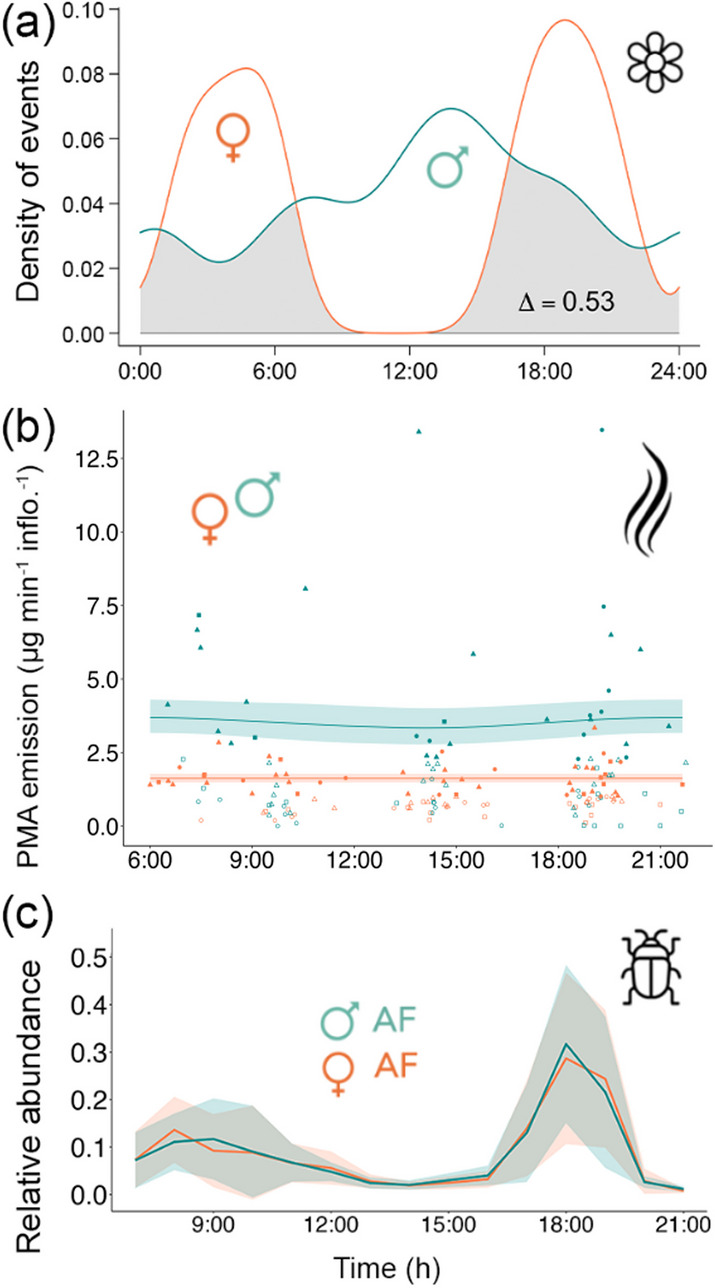
Inflorescence opening, odor release, and insect visitation patterns in the model *Phytelephas aequatorialis.* **a** Inflorescence opening. Distribution of opening events during the first day of anthesis for male (*n* = 15, blue curve), and female inflorescences (*n* = 14, orange curve). The overlap coefficient (Δ) is the area under both curves that is shaded in grey. **b** Inflorescence scent. Generalized additive model (GAM) curves of the release time of the volatile compound p-methylanisole in female (*n* = 89) and male (*n* = 80) inflorescences. The solid line of the GAM model is the predicted average amount of p-methylanisole released at each time of day and the shaded area indicates the 95% confidence interval of the prediction. The solid dots of the GAM model represent 75% of the maximum p-methylanisole scent emission captured in female and male inflorescences used to construct the model (*n* = 48 for females and *n* = 31 for males). The empty dots in the GAM model represent individual odor measurements collected from all sampled inflorescences (*n* = 89 for females and *n* = 80 for males). Each point corresponds to an independent odor sampling in the inflorescences. The shape of the dots represents the day from the anthesis in which we performed the odor sampling. A circle for the first day, a triangle for the second day, and a rectangle for the third day. **c** Visiting insects. Relative abundance of the insect community visiting male (*n* = 12) and female (*n* = 12) artificial flowers throughout the day*.* The solid lines represent the mean relative abundance per hour and the shaded area is the standard deviation per hour

The male inflorescences of *P. aequatorialis* opened without a defined time of day, despite a slight peak in flowering frequency occurring from afternoon to early evening (Fig. 2A). In contrast, the female inflorescences exhibited a clearly bimodal opening pattern, with two well-separated peaks: one in the early morning (from as early as 02:00 to 06:00), and another at dusk (16:00 to 21:00; Fig. 2A). The overlap coefficient value of the kernel density curves for the flowering events of both sexes was 0.53 (95% CI = 0.30 to 0.74), indicating that there is on average 53% probability that female and male inflorescences opened at the same time of the day (Fig. 2A).

### Emission of Volatile Organic Compounds (VOCs)

We compared the data of maximum odor releases over a day in male and female inflorescences in the first days of anthesis (Fig. 2B). There was a significant difference in odor-released quantity between male and female inflorescences in the samplings we carried out every 3 h at different times throughout the day (intercept *P* = 7.39e-16). Male inflorescences released a maximum of 13.47 μg min^−1^ inflorescence^−1^ and a mean of 4.70 ± 3.38 μg min^−1^ inflorescence^−1^ of p-methylanisole, compared to the maximum of 3.34 μg min^−1^ inflorescence^−1^ and the average of 2.12 ± 0.51 μg min^−1^ inflorescence^−1^ of p-methylanisole of female inflorescences (Fig. 2B). For both female and male inflorescences, we found no differences in the diurnal variation of odor emission throughout the day (Fig. 2B, GAM, *p* = 0.76 and 0.20, respectively). In other words, there was no change in the amount of odor released per minute throughout the day in either sex. Our five-variables GAM model explained 68% of the variation in odor emission for both sexes (*R*^2^ = 0.68; formula in the Online Resource 2) with time after anthesis identified as the most significant variable (for further details see Online Resource 2).

### Daily patterns of visiting insects (experiment #1 daily mimicry)

Overall, the male and female AF attracted a comparable cumulative number of insects at the end of each day (5,703.71 ± 3,630.33 in male AF compared to 1,837.14 ± 695.64 in female AF; Kruskal–Wallis *P* = 0.14; Post Hoc Z = 1.67). The control treatment captured a much smaller cumulative number of insects at the end of the day (8.5 ± 4.97) compared to male AF and female AF (Kruskal–Wallis *P* = 1.83e-04, 0.03; Post Hoc Z = −3.84, −2.24, respectively). The results of the GML model indicate a significant increase in the number of insects captured in female AF, with an even greater effect observed in male AF (*P* < 2e-16 for both AFs) compared to the control group. The daily patterns of the relative abundance of the visiting insect community were similar between female AF and male AF, with two peaks: one in the early morning around 8:00 for female AF and 09:00 for male AF and a second at 18:00 for both AFs. We recorded minimum abundances at 14:00 and 21:00 for both AFs (Fig. 2C).

**Fig. 3:**
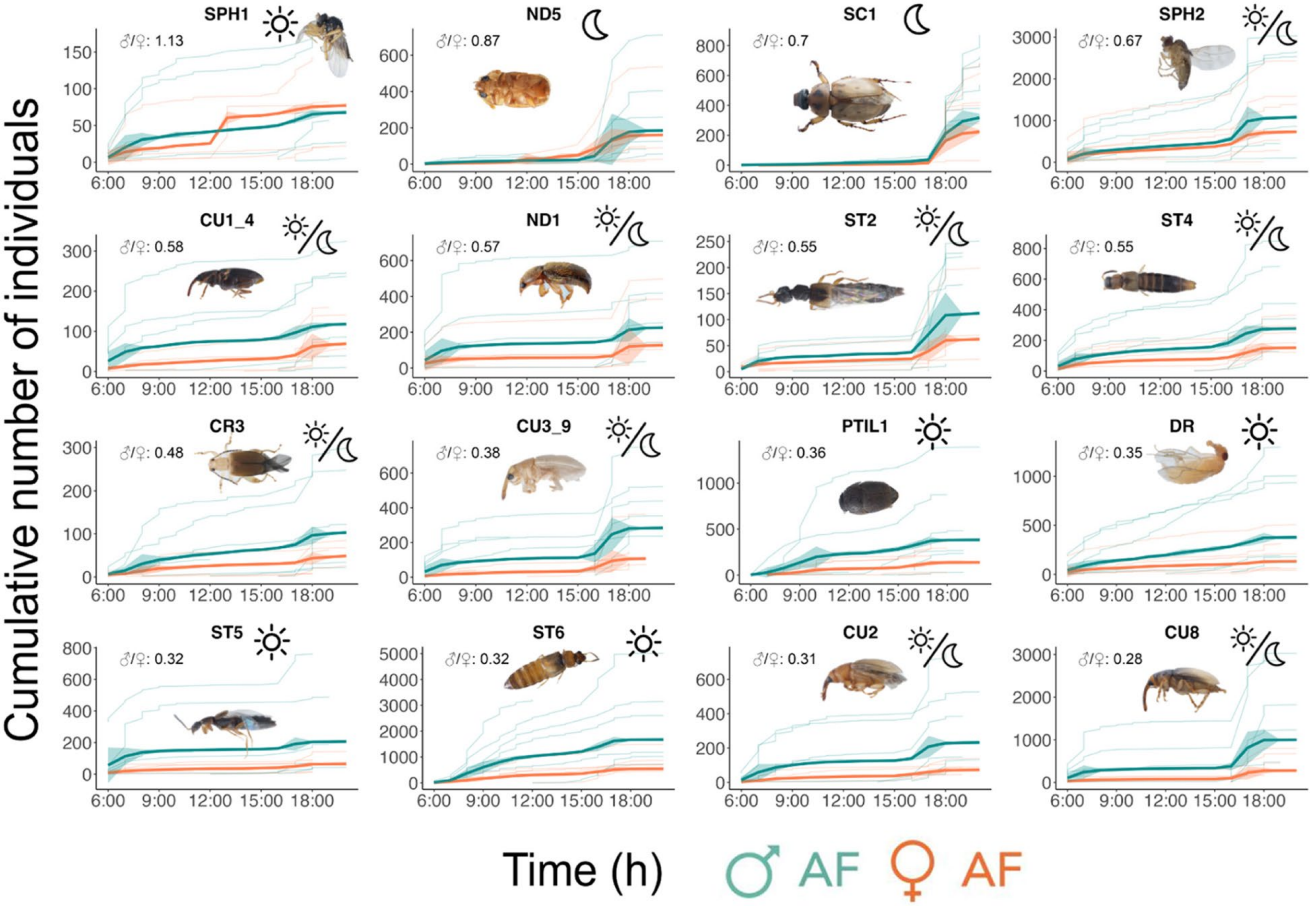
Daily cumulative abundances of the most abundant insects collected using male and female artificial flowers of *Phytelephas aequatorialis*. The transparent lines represent the 12 repetitions of the experiments, the thick lines are the mean abundance of insects sampled per hour and the shaded area is the standard deviation per hour. The ratio value is the difference between the total number of insects captured in male AF (♂ = 400 μL of p-methylanisole) and female AF (♀ = 50 μL of p-methylanisole). The 16 selected morphospecies represent 90% of the total abundance of insects captured for both female and male AFs. Graphs are ordered from maximum to minimum ratio value (indicated in the top-left corner for each vignette). The symbols in the upper right corner of each chart correspond to the habit, sun = diurnal, moon = nocturnal, sun/moon = vespertine. The acronym of each morphospecies corresponds to the taxonomic family to which they belong: Coleoptera: CR Chrysomelidae, CU Curculionidae, ND Nitidulidae, SC Scarabaeidae, ST Staphylinidae, PTIL Ptilidae, Diptera: DR Drosophilidae, SPH Sphaeroceridae. The numbers after the taxonomy abbreviations generate a unique code in the order in which we define the different morphospecies found for each family. Figure S6 in the Online Resource 3 contains the same information but with a different representation

When examining the activity patterns of the 16 most abundant morphospecies (Fig. 3), we found that nine morphospecies exhibited vespertine behavior (activity at dawn and dusk), including eight Coleoptera (namely CR3, CU2, CU3_9, CU8, CU1_4, ND1, ST2, and ST4) and one Diptera (SPH2). The remaining five morphospecies were diurnal (activity mainly in the morning and throughout the day), referring to two Diptera (DR and SPH1) and three Coleoptera (PTIL1, ST5, ST6). The last two morphospecies were nocturnal Coleoptera, namely SC1 and ND5 (see further details in the Online Resource 3). Furthermore, ten morphospecies (CR3, CU2, CU3_9, CU8, ND1, PTIL1, ST4, ST5, ST6) exhibited significantly reduced daily abundances in female AF compared to male AF (Kruskal–Wallis *P* < 0.05; Post hoc Z-statistic > 0, see values in Table 1). However, when accounting that female inflorescences stay open twice as long than males, only one insect morphospecies (Diptera DR) exhibited statistically higher abundances in males than in female AF (Post hoc Z-statistic > 0, see values in Table 1). In summary, over the course of two days, female AFs attracted a similar number of insects in 15 out of the 16 most abundant insect species, compared to the number of insects collected in a single day from male AFs.

**Table 1:** Kruskal Wallis *P* and post hoc Z statistic comparison of the number of insects captured in one-hour intervals from 06:00 to 21:00 in female and male artificial flowers for the 16 most abundant visiting morphospecies

Morpho-species	Male AF one day—Female AF one day		Quantity comparison	Male AF one day—Female AF two days		Quantity comparison
	***P***	***Z***		***P***	***Z***	
CR3	1.44E-05*	4.34	**Male AF** > Female AF	0.88		**Male AF = Female AF**
CU2	7.66E-04*	3.36	**Male AF** > Female AF	0.89		**Male AF = Female AF**
CU3_9	9.43E-04*	3.30	**Male AF** > Female AF	0.98		**Male AF = Female AF**
CU8	0.01*	2.65	**Male AF** > Female AF	1		**Male AF = Female AF**
CU1_4	0.12		**Male AF = Female AF**	0.03*	−2.17	Male AF < **Female AF**
DR	4.15E-09*	5.88	**Male AF** > Female AF	0.05*	1.97	Male AF > **Female AF**
SPH1	0.15		**Male AF = Female AF**	0.01*	−2.59	Male AF < **Female AF**
SPH2	0.05		**Male AF = Female AF**	0.08		**Male AF = Female AF**
SC1	0.82		**Male AF = Female AF**	0.01*	−2.59	Male AF < **Female AF**
ND5	0.99		**Male AF = Female AF**	3.13E-04*	−3.60	Male AF < **Female AF**
ND1	2.07E-03*	3.08	**Male AF** > Female AF	0.97		**Male AF = Female AF**
PTIL1	0.01	2.60	**Male AF** > Female AF	0.78		**Male AF = Female AF**
ST2	0.09		**Male AF = Female AF**	0.21		**Male AF = Female AF**
ST4	8.85E-04*	3.32	**Male AF** > Female AF	0.58		**Male AF = Female AF**
ST5	0.02	2.42	**Male AF** > Female AF	0.46		**Male AF = Female AF**
ST6	1.37E-07*	5.27	**Male AF** > Female AF	0.12		**Male AF = Female AF**

^Male AF (artificial flower with 400 μL of p^^−^^methylanisole). Female AF (artificial flower with 50 μL of p^^−^^methylanisole)^

^One day refers to the average insect capture values in a one^^−^^day time series^

^Two days refers to a*2 multiplication of the mean insect catch values in a one^^−^^day time series, resembling two days, simulating the behavior of a female inflorescence that stays open longer^

^Asterisks represent a significant*P* (< 0.05) in the Kruskal–Wallis test indicating a significant difference in the mean abundance between the two groups^

^Z is the statistic that indicates the magnitude of the standard difference between the groups compared^

^Quantity comparison reflects if male AF attracts a higher number of insects than female AF or vice−versa^

^The name of each morphospecies corresponds to the taxonomic family to which they belong: Coleoptera: *CR*Chrysomelidae, *CU*Curculionidae, *ND*Nitidulidae, *SC*Scarabaeidae, *ST*Staphylinidae, *PTIL*Ptilidae, Diptera: *DR*Drosophilidae, *SPH*Sphaeroceridae^

^The numbers after the taxonomy abbreviations generate a unique code in the order in which we define the different morphospecies found for each family^

^The AF with the highest and significantly higher number of attracted insects is marked in bold. Where both treatments attracted statistically similar numbers the two are in bold^

It is noteworthy that the abundance of AF-visiting insect morphospecies exhibited considerable fluctuations throughout the day, despite the constant release of odor by the AFs. Only CR3, SPH1, PTIL1, and ST5 exhibited a similar abundance of visits to the female AFs across the day. For the 16 morphospecies, between-hour differences in insect abundance were statistically higher in male AFs compared to female AFs (Kruskal–Wallis *P* = 0.01; Post hoc Z-statistic = 2.49; Online Resource 3 for more details). Despite the smaller emission of p-methylanisole, female AFs attracted a more constant number of insects than male AFs.

### Early visiting insects (experiment #2 early attraction)

**Fig. 4:**
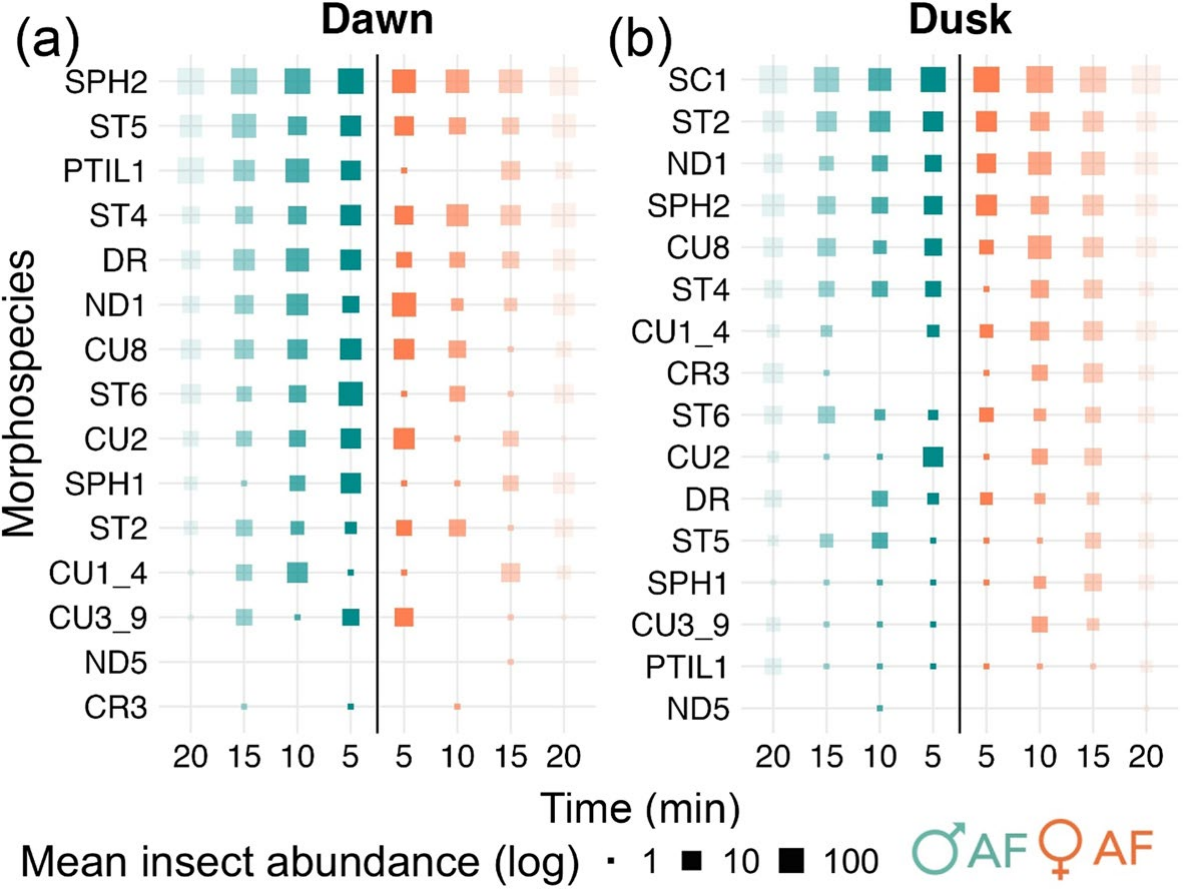
Mean number of early visitors caught every five minutes at three-time intervals at (**a**) dawn and (**b**) dusk, using artificial flowers of *Phytelephas aequatorialis*. The orange squares represent the bioassays using female AF (time series = 6) and the blue squares the bioassays using male AF (time series = 6). The solid color is in the squares representing the number of insects at five minutes and it becomes transparent up to 20 min. The log-transformed mean insect abundances are represented on a logarithmic scale with base 10. Specifically, log₁₀(1) = 0, log₁₀(10) = 1, and log₁₀(100) = 2, with each increase of 1 representing a tenfold rise in insect abundance. The 16 selected morphospecies represent 90% of the total abundance of insects captured for both female and male AFs. The acronym of each morphospecies corresponds to the taxonomic family to which they belong: Coleoptera: CR Chrysomelidae, CU Curculionidae, ND Nitidulidae, SC Scarabaeidae, ST Staphylinidae, PTIL Ptilidae, Diptera: DR Drosophilidae, SPH Sphaeroceridae. The numbers after the taxonomy abbreviations generate a unique code in the order in which we define the different morphospecies found for each family

Overall, male and female AFs attracted a similar number of insects over each 20-min collecting period (38 ± 39.08 in male AF vs. 33.89 ± 41.07 in female AF; Kruskal–Wallis *P* = 1; Post Hoc Z = 0.34). The control treatment captured a much lower number of insects (1.4 ± 0.52; Kruskal–Wallis *P* = 3.35e-05, 1.12e-04; Post Hoc Z = −4.24, −3.96, in comparison to male and female AFs, respectively). At both dawn and dusk, 14 different morphospecies arrived at the female and male AFs as early as five minutes after the start of the experiment (Fig. 4). We found no significative difference in the mean abundance of insects attracted to AFs in the five-minute samples, when grouped or blocked by the amount of p-methylanisole applied (50 μL for female AFs and 400 μL for male AFs), at either dawn or dusk (Quade test *P* = 0.052 and 0.12, respectively). The most abundant morphospecies after five minutes (and up to 20 min) were SPH2 (Sphaeroceridae, Diptera) and SC1 (Scarabaeidae, Coleoptera) in male and female AFs at dawn and dusk, respectively (for details see Online Resource 3).

## Discussion

The objective of this study was to determine the daily patterns of inflorescence opening, odor release, and insect visitation with high temporal resolution in *P. aequatorialis*. We then aimed to analyze sex-specific differences in these traits within a pollination system maintained through intersexual mimicry. We show that the subtle differences in floral traits between the two sexes do not alter the visitation rates in mimicking females. Subsequently, we discuss how the results of our experiments with AFs support the “non-preference” hypothesis.

In dioecious systems, it has been suggested that the opening and release of floral odors are temporally restricted to the time of natural activity of pollinators in both sexes [40]. Our study presents coordinated dawn- and dusk-time female opening, while male opening can happen anytime for *P. aequatorialis* (Fig. 2A). The observed bimodal female inflorescences phenology is consistent with the findings that female ‘flowers’ in dioecious plants exhibit high levels of synchrony throughout the day, meaning a greater proportion of flowers open simultaneously within the same inflorescence [41]. Female flowers in dioecious models also remain open up to three or four days thus increasing diel overlap with pollinator activity [41]. The opening of female inflorescences may be synchronized with the foraging time window of insects moving between male inflorescences. The extension of anthesis over several consecutive days may help maximize ovule fertilization [42]. Therefore, we suggest that females are under stronger selection than males to display such synchronization in their opening. A less synchronized daily flowering pattern in male inflorescences may be attributed to several factors, including an intrasexual competition to fertilize ovules, the necessity for a constant supply of pollen for pollinators with diverse habits (e.g., diurnal, nocturnal, and so forth), or differences in resource allocation between sexes [14]. Within the subfamily Phytelephantoideae, the discrepancy in the daily flowering patterns of the sexes may influence positively the movement of pollinators from pollen-producing male flowers with a shorter lifespan to female flowers with greater longevity [43].

Another dimorphic trait observed in *P. aequatorialis* is the release of higher amounts of p-methylanisole by male inflorescences compared to females (Fig. 2B). Similar to what we have documented here for *P. aequatorialis*, no variations in the amount of VOCs were reported in the dioecious dwarf palm (*Chamaerops humilis)* during different phenological phases [44]. The absence of a daily odor-release pattern from the inflorescences of both sexes of *P. aequatorialis* may be a means of maximizing odor release during the anthesis of short duration in both sexes [41]. Alternatively, it may be indicative of a physiological adaptation of the flowers to insects with diverse habits and visitation periods, as observed by [45] in flower visitation networks. A constant odor release throughout the day could enable *P. aequatorialis* to target a variety of species, including both specialists and generalists responsive to p-methylanisole, with different activity patterns, thereby increasing the likelihood of pollination [20], particularly since *P. aequatorialis* occurs in diverse environments and blooms only in the dry season. This increases the likelihood of attracting large numbers of pollen-laden insects from male to female inflorescences by prolonged opening and release of attractants such as odor, even though the insects may be deceived and visit after only 5 min.

The present study demonstrates that the diel activity of visiting insects is independent of the amount of odor used to attract them, when simulating constant odor release at different amounts of p-methylanisole as we found to occur in real inflorescences (Fig. 3). Previous studies on pollinator communities and specialized plant–pollinator interactions have also reported bimodal floral foraging patterns and 24-h rhythms in olfactory stimulus receptivity [4, 32]. This highlights the importance of conducting pollination ecology studies at a daily temporal scale, incorporating both internal and external environmental factors that extend beyond the limitations of the present study, to better understand the complexity of ecological interaction dynamics [1].

The findings obtained with the AFs in the Experiment #1 Daily Mimicry are in accordance with the evidence that the Coleoptera and Diptera orders are the most predominant taxa attracted to a p-methylanisole source [46]. Nevertheless, our daily scale study with AFs corroborates the importance of a fly from the family Sphaeroceridae (SPH2) as an early and abundant visitor attracted by the main VOC of *P. aequatorialis* floral scent [47]. This family has been reported as a rare pollinator of angiosperms and has been specifically reported to pollinate for Araceae [48]. Previous studies in palm pollination have focused on Coleoptera [20] and the nursery mutualism involving Curculionidae and Nitidulidae [49]. Overall, the visiting insect community captured with AFs included both male and female individuals of insects, just as in real inflorescences. As part of a personal observation, we enclosed two male inflorescences of *P. aequatorialis* in individual transparent cloth covers and monitored insect activity overnight. In light of our findings, we propose that real inflorescences serve as reproductive hubs for insect species of both sexes. Our observations support this claim, as we repeatedly recorded individuals of several Coleoptera families (e.g., conspecific pairs of Chrysomelidae) copulating on male inflorescences, and even conspecific pairs of a Scarabaeidae species copulating on the AFs. In addition, one species of Drosophilidae (Diptera) showed conspecific exclusion behavior on the AFs, suggesting a form of territoriality. However, confirming this claim requires a focused study aimed at identifying and quantifying the frequency of conspecific insects copulating within male and female inflorescences.

The results of our experiments support the “no-preference” hypothesis of deceptive pollination, which postulates that pollinators are unable to distinguish between male and female inflorescences. Previous studies of dioecious systems have proposed that for unrewarding female inflorescence with a low frequency of visits, synchronizing the odor emission with the timeframe of higher pollinator activity can enhance encounters [50]. However, our results indicate that there is no discernible pattern of variation in scent emission throughout the day in either sex (Fig. 2B) despite the insect activity appears to be mostly bimodal with higher activity at dawn and dusk. Interestingly, we observed a bimodal pattern of bract opening in female inflorescences (some opening in the early morning and others in the evening) coinciding with these peaks in insect activity. This temporal overlap may indicate a sequential presentation of newly opened unvisited floral tissues that contribute to pollinator attraction.

The artificial flower demonstrated that both male and female AFs of *P. aequatorialis* exhibited comparable richness and abundance of insect attraction at different times of the day (Fig. 2C). This may indicate that floral mimicry exploits the sensory biases of floral visitors [51]. Previous hypotheses suggest that insects have an innate preference for mimicry, but with experience, insects may learn to associate only male inflorescences with reward (e.g., mating or shelter), potentially leading to reduced visits to female inflorescences over time [15]. However, the similar attractiveness of flower visitors observed in our study suggests that potentially naive insects respond indiscriminately to both sexes. Moreover, the similarity in visitor numbers is more pronounced when the longer opening period of the female inflorescences of *P. aequatorialis* is taken into account (Table 1), thereby increasing the probability of deceiving a diverse insect community. As the female inflorescence remains open for a longer period of time than the male inflorescence (Auffray T, Beugnon R, Orellana G, Guasti A, Pincebourde S, Montúfar R, Dangles O, Gibernau M: Hot cheating: intersexual mimicry underlies efficient pollinator attraction in a dioecious thermogenic palm, unpublished), it is reasonable to suggest that the female receives a greater number of visitors at the end of its life cycle than the male inflorescence. The high frequency of pollinators at both odor sources of p-methylanisole in the AFs suggests that the lower p-methylanisole emission rate of female inflorescences may not allow insects to discriminate according to tested p-methylanisole amount, explaining the high visitation rate to the mimicking sex, i.e. females [16, 19]. This finding calls into question the prevailing hypothesis that a greater stimulation of olfactory receptors in insects drives the preference for a strong odor source, resulting in a higher visitation rate to males [52].

## Conclusion

The collective findings provide evidence for the absence of preference by insect visitors for either source of p-methylanisole when assessed over a daily and a fine-5-min timescale (Fig. 3 and 4). The results indicate that after only five minutes, the olfactory mimicry deception was effective in attracting the same insect groups to male and female AFs, despite the difference in the amount of the volatile p-methylanisole released. In light of the existing literature, we propose that early visitation can serve as a potential indicator of pollination efficiency and the success of deception [53]. However, without direct measurements of pollination success or seed set, the relationship between visitation rates and actual pollination outcomes remains to be fully demonstrated. This relationship may nonetheless be particularly relevant for female inflorescences that don’t offer reward and have limited amounts of ovules, as the first pollen visits could determine the fruit set [54]. Taken together, these results highlight the importance of early visitation and suggest why unrewarded deception may have been evolutionarily maintained as a pollination mechanism.

## Supplementary Information

Supplementary Material 1.

Supplementary Material 2.

Supplementary Material 3.

## Data Availability

The datasets generated, and analyses performed during the current study are available in the Zenodo Digital Repository, 10.5281/zenodo.14452502.
