# Supplementary material for: Experimental evidence of pollination by deception in a dioecious palm
Source: BMC Ecol Evol. 2025 May 12;25:46. doi: 10.1186/s12862-025-02388-6 (PMC12067733; doi:10.1186/s12862-025-02388-6)
Supplement: Supplementary file 1 — Supplementary Material 1. [file 12862_2025_2388_MOESM1_ESM.docx]

**Experimental evidence of pollination by deception in a dioecious palm**

Galilea Orellana-Vera^1^, Thomas Auffray^2^, Rommel Montúfar^1^, Marc Giberneau^3^, Sylvain Pincebourde^4^, Arturo Guasti^5^, Jérôme Casas^4^ and Olivier Dangles^1,2^

*^1^ PUCE, Facultad de Ciencias Exactas y Naturales, Quito, Ecuador, ^2^ CEFE, Université de Montpellier, CNRS, EPHE, IRD, Montpellier, France, ^3^ LSPE, CNRS, Université de Corse, Ajaccio, France, ^4^* *IRBI, CNRS, Université de Tours, Tours, France, ^5^OTONGA Foundation, Quito, Ecuador*

**Electronic supplementary material (ESM)**

**Online Resource 1 – Artificial flower**

1. **Description of the device**

The utilization of the artificial flower permitted the dissociation of the natural daily phenology of a dioecious model with odor serving as the sole component of variation. Furthermore, the utilization of external biomimetic flowers in ecological experiments presents a distinct advantage, as it eliminates the potential for bias resulting from the assumption that insects have learned the location of flowers and will inevitably return to them, even after manipulation, if this had been the case [1]. P-methylanisole has been identified in the floral scent of 17 additional plant families [2, 3], yet it accounts for 99% of the whole chemical profile of *P. aequatorialis* inflorescences [4], and one uncommon VOC in higher proportions acts as a private channel providing specificity to the attracted insect community, just as described for *Ficus semicordata* [2, 5]. Furthermore, the combination of a few synthetic compounds has been shown to effectively attract specific pollinators of real flowers, as demonstrated for *F. carica* [6,7]. This approach allows us to test previously untested hypotheses regarding dioecy and deceptive pollination [1]. Although there are numerous methods for measuring pollination efficiency [8-12], for the purposes of this study, we have chosen to compare the different treatments in terms of visit frequency, as reflected by abundance.

We utilized an interception trap baited with p-methylanisole (referred to as the “artificial flower”) to ascertain the daily insect activity. Although few in number, other studies have used artificial scent baits to investigate pollination and factors influencing the daily activity of insects [5,11,13]. The artificial flower comprises two units: an attractive unit and a trapping unit (Fig. S1). The attractive unit is designed to simulate the interaction between the volatile organic compound (VOC) and floral thermogenesis. We affixed a Petri dish to a Peltier module. The Petri dish serves to diffuse the VOCs, while the Peltier module provides heat generation. The temperature of the module is meticulously regulated via a DC-DC buck converter, which modulates the voltage provided by a 9V lithium battery. The tip of a Type T copper/constantan beaded thermocouple sensor (±0.1°C precision; Onset Computer Corporation, Bourne, USA) connected to a HOBO 4-channel thermocouple datalogger (Onset Computer Corporation, Bourne, USA) measured the diffuser temperature continuously, while a second sensor measured the ambient air temperature. We adjusted the temperature of the module to 10 °C above the ambient temperature, to simulate the temperature excess observed in both male and female inflorescences at the time of opening. The trapping unit collected visitor insects. It comprised a transparent 5L plastic bottle inverted with the neck facing downwards, with two large cut openings facing each other, and one vertical perpendicular transparent panel parallel to the openings positioned at the center of the bottle. The attractive unit was maintained in a position below the panel. The attracted insects collided with the panel and fell into a container that was screwed to the bottle cap. The container was filled with water and an odorless soap solution in order to prevent the trapped insects from escaping. We established the following insect capture sequence. We deposited a specific quantity of VOC into the diffuser apparatus via a 100 µL Hamilton syringe after which we powered the module by means of a 9V battery. Following a period of 50 minutes, we collected the container with trapped insects and replaced it with a new container. We also replaced the battery with a fully charged battery and renewed the VOC after wiping it with a paper towel.


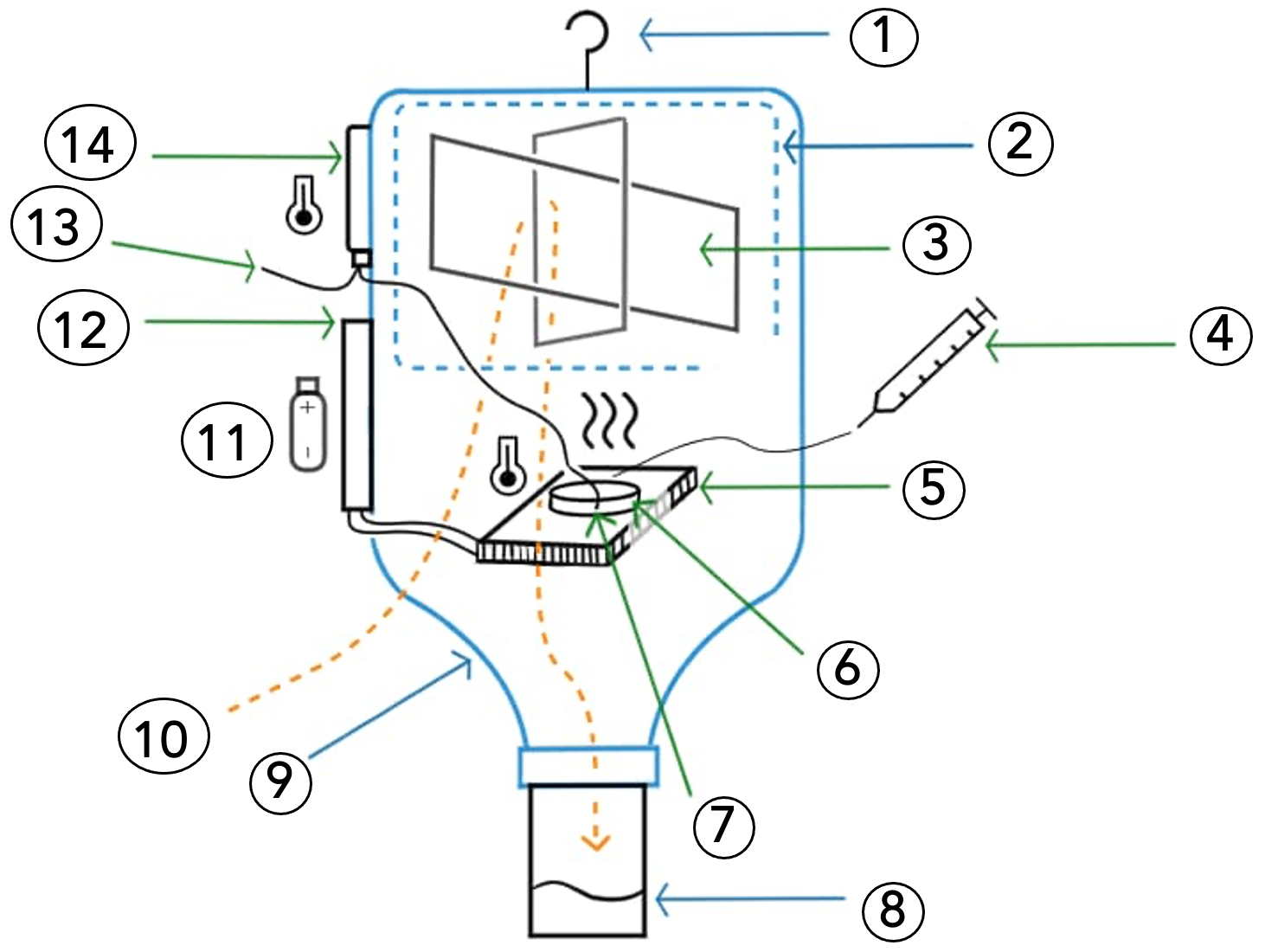


Fig. S1 Diagram of the Artificial Flower. In blue the structural parts of the trap, in green the odor and heat components, and in yellow the interception performance. (1) Suspension hook, (2) bottle opening, (3) perpendicular transparent panel, (4) syringe with p-methylanisole, (5) Peltier module, (6) petri dish, (7) thermocouple sensor (diffuser), (8) container with water and odorless soap, (9) 5L plastic bottle, (10) insect path, (11) 9V lithium battery, (12) DC-DC buck converter, (13) thermocouple sensor (air temperature), and (14) HOBO 4-channel thermocouple datalogger

**2. Comparison of insect community composition between real inflorescences and artificial flowers**

Due to the large difference in shape and structure of the surroundings, it is likely that the aerodynamic environment, and consequently the behavior and diffusion of odors, will differ between real inflorescences and artificial flowers. However, our aim in using artificial flowers in this study was not to physically mimic real inflorescences but to have a manipulative system that could mimic the attraction of insects to real inflorescences. To ascertain the reliability of the results obtained from the artificial flower and to provide support for its utility, we compared insect capture data from real inflorescences and artificial flower experiments (using 200 μL of p-methylanisole in the AFs), which were carried out simultaneously in 2021 within the forest of the Otongachi Ecological Reserve.

Firstly, we compared the composition and abundance of the insect community attracted to both artificial flowers and real inflorescences of *P. aequatorialis* using non-metric multidimensional scaling (NMDS). Due to high variation in the sampling duration between artificial flowers and the true inflorescences (1h vs 3h to 72h), we report the abundance of insects collected by sample to relative abundance per hour. Given that insects captured on true inflorescences were not captured continuously during the whole trapping duration due to their specific periods of activity (for example, on a 24h trapping duration, ND4 species can be captured only during the interval 17h-21h according to the activity pattern of this species), we extract the real duration during which an insect species can be effectively trapped on inflorescences during a trapping sequence. For that, we first modeled the activity pattern obtained from the 1h-scale artificial flower for each species using a zero-inflated negative model with negative binomial distribution using the function *glmmTMB* from the package “glmmTMB,” including the number of captures as the response variable and the insect species identity interacting with the time of the day as predictive variables. The date and the site of the experiment were used as random factors, and the zero part of the model was modeled with the species, time of the day, and date (i.e., the absence of captures can depend on the addition of the species, the time of the day and the date). According to the model pattern, we extracted the time boundary of each peak of activity. For that, we used a kernel density, with bandwidth set to 1 hour to identify the time when captures were the most abundant. Then, we calculated the total area under the curve, and we extracted the activity peaks by selecting the points of the density curve above a threshold = 0.95% (i.e. we selected the part of the time series in which 95% of the abundance of one species occurs), and we extracted the time of these peak boundaries. Finally, in each insect sample collected on inflorescences, we divided the abundance of each species captured by the duration during which that species may have been active, calculating the duration of its period of activity within the sampling interval on the inflorescences, allowing us to obtain a number of captures / h for each species. Due to the high number of zeros captures per hour in the samples when no insect activity was present, the NMDS was run with Hellinger distances using the “base” and “vegan” packages in R language [14,15]. We found that the insect composition was significantly different between real inflorescences and the artificial flower (Permanova df = 435, R² = 0.84, F= 78.6, *P* = 0.001***; Fig. S2).


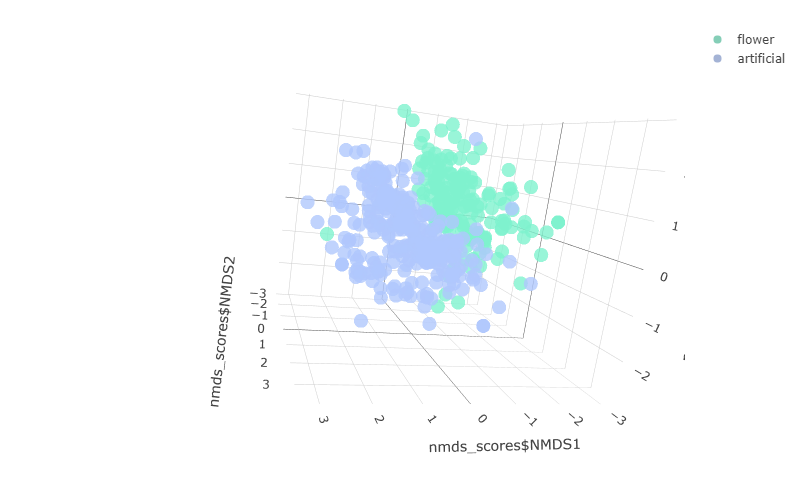


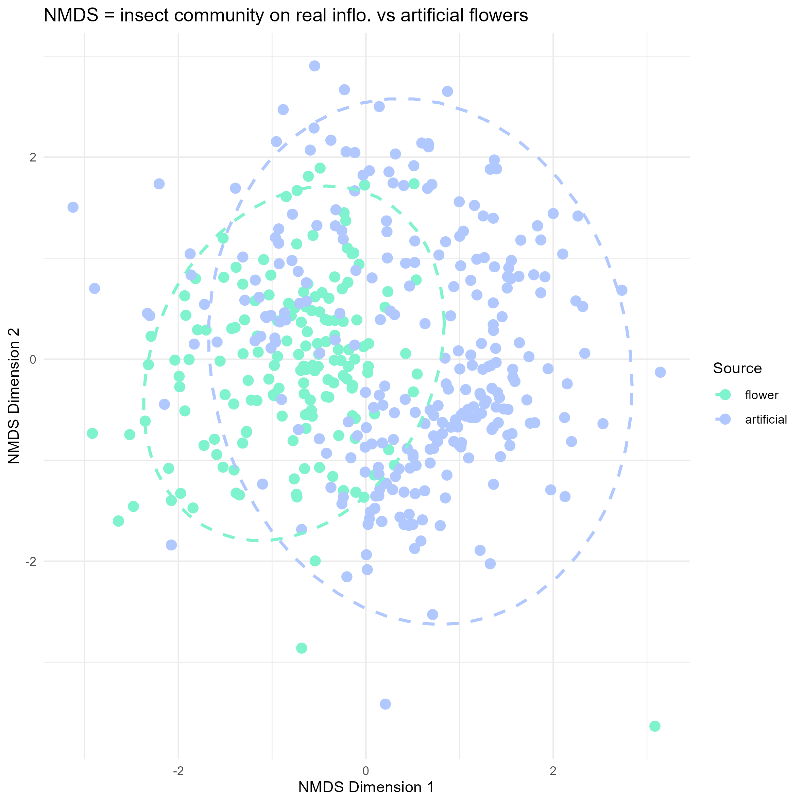


Fig. S2 Non-metric multidimensional scaling analysis (NMDS, we set k = 3 because the stress value was too high with k =2) of the insect visiting community in artificial flowers and true inflorescences of *Phytelephas aequatorialis*. The left panel shows a 2D representation with ellipses of the first two axes (respective variance = 1.33 and 1.12), and the right panel is a 3D representation obtained with the package “plotly” on R (variance of the third axe = 0.99). Samples collected from real inflorescences are represented by green dots, and samples collected from artificial flowers are blue dots. The stress value is 0.18

Due to the discrepancy in data collection methodology between artificial flowers and real inflorescences explained before, we proceeded to compare the principal morphospecies attracted to each type of flower, given that our analyses concentrate on the most numerous morphospecies attracted to p-methylanisole sources (Fig. S3). A comparison of the percentage contribution of each morphospecies revealed that both types, real inflorescences and artificial flowers, attract the same main morphospecies and that the communities have a similar composition. Due to the high variability in the abundance of the insect community in each treatment, no significant difference was found in the mean quantity of the main morphospecies, except for ST5 and ND1 (Fig. S3).

**

**

**Fig. S3 Bar chart of (a) the individual contribution percentage and (b) the average number of the most abundant morphospecies that visit artificial flowers and real inflorescences of *Phytelephas aequatorialis*.** The green bars represent the contribution percentage of morphospecies with interception traps placed in front of female and male inflorescences of *P. aequatorialis* for time intervals of four to six hours. Blue bars represent the contribution percentage of morphospecies captured in artificial flowers (AF) with one-hour samplings grouped in 4-hour intervals throughout the day. For real inflorescences, 16 morphospecies (except for SYLV1, ND2, CU1_4, and CR3) represent 89.12% and in artificial flowers, 16 morphospecies (except for CU8, HYMD, HYM2, and SCO1) make up 99.03%. Asterisks represent a significant *P* in the Kruskal-Wallis test indicating a significant difference in the mean abundance between the two groups (ST5 *P* = 0.003, ND1 *P* = 0.047). The name of each morphospecies corresponds to the taxonomic family to which they belong: Coleoptera: CR = Chrysomelidae, CU = Curculionidae, Histeridae = HIST, ND = Nitidulidae, SC = Scarabaeidae, Scolytinae = SCO, ST = Staphylinidae, PTIL = Ptilidae, Diptera: DR = Drosophilidae, SPH = Sphaeroceridae, Hymenoptera = HYM. The numbers after the taxonomy abbreviations generate a unique code in the order in which we define the different morphospecies found for each family

**3. Setting the amount of p-methylanisol in male and female artificial flowers**

To determine the ratio of p-methylanisole amounts in female vs male AF in our experimental study, we considered mimicking the competitive interaction between female AF with lower p-methylanisole sources (equivalent to *P. aequatorialis* female inflorescences open for several days) and male AF with higher odor sources (equivalent to newly opened *P. aequatorialis* male inflorescences at peak emission). Fig. S4 shows that this ratio is on average 1:8, it comes from the 25% of the highest points in males D+1 = 5.34 µg min^-1^ flower^-1^, and between the first and third quartile in females D+1 (25% to 50%) of the points in females (not 0-25% to discard outliers) = 0.61 µg min^-1^ flower^-1^. Multiplying by one hour or 60 minutes gives 320µL for males and 36µL for females. For more convenience, and to avoid handling errors during odor injection in a Petri dish, we have rounded off these amounts to 400µL and 50µL (as a minimum amount to avoid rapid drying) of p-methylanisole, to correspond to a male with a high emission, and therefore very competitive, and a slightly more than the average female. As a result, we used 50 μL of p-methylanisole for female AF and 400 μL of p-methylanisole for male AF.


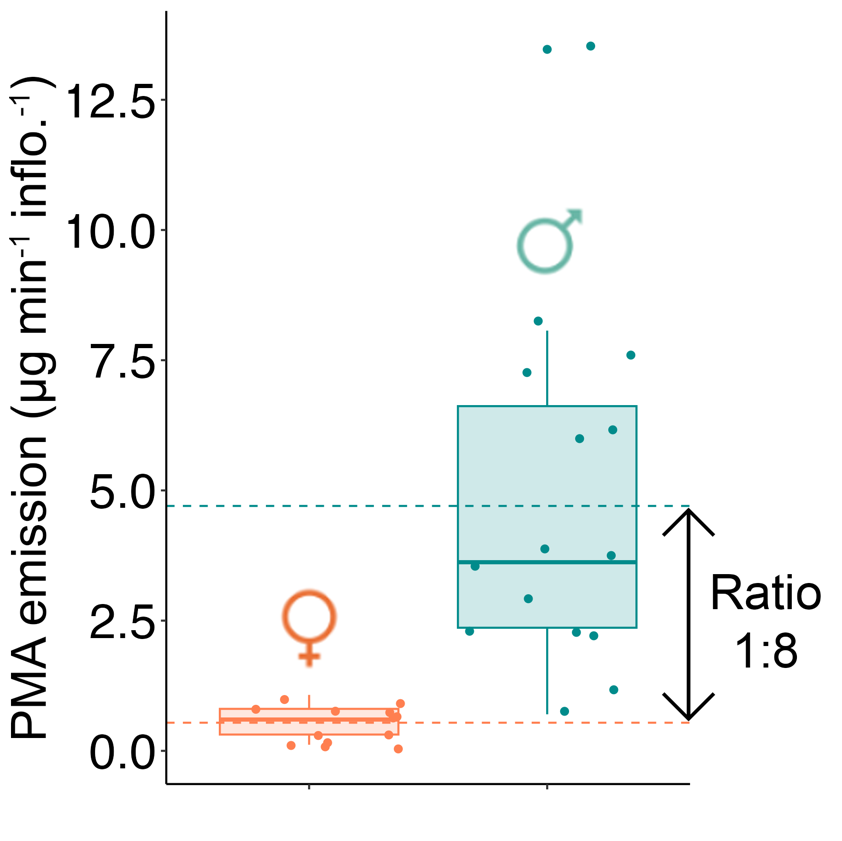


Fig. S4 Amount of p-methylanisole released in each inflorescences sex sampled of *Phytelephas aequatorialis*. The blue dots in the boxplot represent the maximum odor release values for each sampled male natural inflorescences (n = 15) resembling the maximum yield and the orange dots in the boxplot are the minimum odor release values in each sampled female natural inflorescences resembling the lowest yield (n = 13)

**REFERENCES**

[1] Ashman TL. Pollinator selectivity and its implications for the evolution of dioecy and sexual dimorphism. Ecology 2000; 81: 2577–2591.

[2] Chen C, Song Q, Proffit M, et al. Private channel: a single unusual compound assures specific pollinator attraction in Ficus semicordata. Functional Ecology 2009; 23: 941–950.

[3] Knudsen JT, Eriksson R, Gershenzon J, et al. Diversity and Distribution of Floral Scent. The Botanical Review 2006; 72: 1–120.

[4] Ervik F, Tollsten L, Knudsen JT. Floral scent chemistry and pollination ecology in phytelephantoid palms (Arecaceae). Pl Syst Evol 1999; 217: 279–297.

[5] Proffit M, Lapeyre B, Buatois B, et al. Chemical signal is in the blend: bases of plant-pollinator encounter in a highly specialized interaction. Sci Rep 2020; 10: 10071.

[6] Gibernau M. Odeurs et spécificité dans les mutualismes figuier-pollinisateur: le cas de *Ficus carica* L. et de *Blastophaga psenes* L. [Doctoral thesis]. Montpellier (France): University of Montpellier; 1997.

[7] Grison-Pigé L, Bessière JM, Turlings TCJ, Kjellberg F, Roy J, Hossaert-McKey MM. Limited intersex mimicry of floral odour in *Ficus carica*. Funct Ecol. 2001;15(4):551–8. doi: 10.1046/j.0269-8463.2001.00553.x

[8] Barker DA, Arceo-Gomez G. Pollen transport networks reveal highly diverse and temporally stable plant–pollinator interactions in an Appalachian floral community. AoB PLANTS 2021; 13: plab062.

[9] De Avila RS, Freitas L. Frequency of visits and efficiency of pollination by diurnal and nocturnal lepidopterans for the dioecious tree Randia itatiaiae (Rubiaceae). Aust J Bot 2011; 59: 176.

[10] King C, Ballantyne G, Willmer PG. Why flower visitation is a poor proxy for pollination: measuring single‐visit pollen deposition, with implications for pollination networks and conservation. Methods Ecol Evol 2013; 4: 811–818.

[11] Knop E, Gerpe C, Ryser R, et al. Rush hours in flower visitors over a day–night cycle. Insect Conserv Diversity 2018; 11: 267–275.

[12] Peralta G, Vázquez DP, Chacoff NP, et al. Trait matching and phenological overlap increase the spatio‐temporal stability and functionality of plant–pollinator interactions. Ecology Letters 2020; 23: 1107–1116.

[13] Armbruster WS, McCormick KD. Diel Foraging Patterns of Male Euglossine Bees: Ecological Causes and Evolutionary Responses by Plants. Biotropica 1990; 22: 160.

[14] Oksanen J, Simpson G, Blanchet F, Kindt R, Legendre P, Minchin P, et al. vegan: Community Ecology Package. R package version 2.6-4. 2022. Available from: <https://CRAN.R-project.org/package=vegan>.

[15] R Core Team. R: A Language and Environment for Statistical Computing. Vienna: R Foundation for Statistical Computing; 2024. Available from: https://www.R-project.org/.

**Online Resource 2 - GAM analysis**

In this study, we present the generalized additive model (GAM) formula, which was used to model the odor release from the female and male inflorescences of *P. aequatorialis* over the course of a single day. This was achieved through the utilization of the *gam* function, which is a part of the “mgcv” R package [1].

$$Scent quantity \sim s\left( collection time, by=sex, bs=c\left( \text{"cc"}, "\text{tp"} \right) \right)+ s(time from anthesis, by= sex, bs=\text{tp") + s(sex, bs = "}re\text{) + s( day since anthesis, by = sex, bs = "re})+ s(collection time, by=flower individual, bs=\text{re") + s(flower individual, by = sex, bs = "}re")$$

(Formula S1)

Where, “collection time”, refers to the time of the sampling (in minutes from 00:00), “sex” refers whether the sampled inflorescences were of the female or male variety, “time from anthesis” refers to the number of minutes elapsed from the time of anthesis, “day since anthesis” denoted the number of days since the day of anthesis, and the term “flower individual” refers to the unique identity code assigned to each sampled inflorescences. Furthermore, the “cc” function is used to set a cubic spline, as time is cyclical and repeats itself. The “tp” function is used for a thin-plate spline or periodic stress, which is used for non-linear relationships. Finally, the “re” function is used for a random effect for each level of the variable which can change the shape of the curve.

**Table S1. Significance of smooth terms of GAM model of odor released of male and female inflorescences of *P. aequatorialis*.**

| **Smooth terms** | **Estimated Degrees of Freedom** | **Reference Degrees of Freedom** | **F-statistic** | ***P*** |
| --- | --- | --- | --- | --- |
| s(collection_minute):sexF | 8,28E-03 | 8 | 0 | 0.76 |
| s(collection_minute):sexM | 7,60E+02 | 8 | 0.19 | 0.2 |
| s(minute_from_anthesis): sexF | 1,00E+03 | 1 | 1.66 | 0.2 |
| s(minute_from_anthesis): sexM | 1,00E+03 | 1 | 10.03 | 0.002** |
| s(sex) | 2,27E-03 | 1 | 0 | 0.44 |
| s(antesis_final):sexF | 2,04E-03 | 1 | 0 | 0.83 |
| s(antesis_final):sexM | 9,90E+02 | 1 | 292.4 | < 2e-16 *** |
| s(flower_code):sexF | 1,90E-03 | 13 | 0 | 0.99 |
| s(flower_code):sexM | 3,79E-02 | 14 | 0 | 0.96 |

^“Collection time”, is the minute of the day when the sampling started, “sex” is either female or male sampled inflorescences, “time from anthesis” represents the minutes from the moment of anthesis, “antesis final” is the number of the day from the day of anthesis, and “flower code” is the identity code of each sampled inflorescence. Asterisks represent a significant^ *^P^* ^(< 0.05) indicating a significant effect of the smooth terms.^

The data indicate that there is no effect on the sex of the inflorescences (*P* = 0.44) or the identity of each sampled individual in the odor emission over time (female *P* = 0.99; male *P* = 0.96). There is a significant difference in the odor emission along the minutes and days when the samples were collected from the time of anthesis in the male inflorescences (minute *P* = 0.002; day *P* = < 2e-16) but not in the female inflorescences (minute *P* = 0.2; day *P* = 0.83).

**
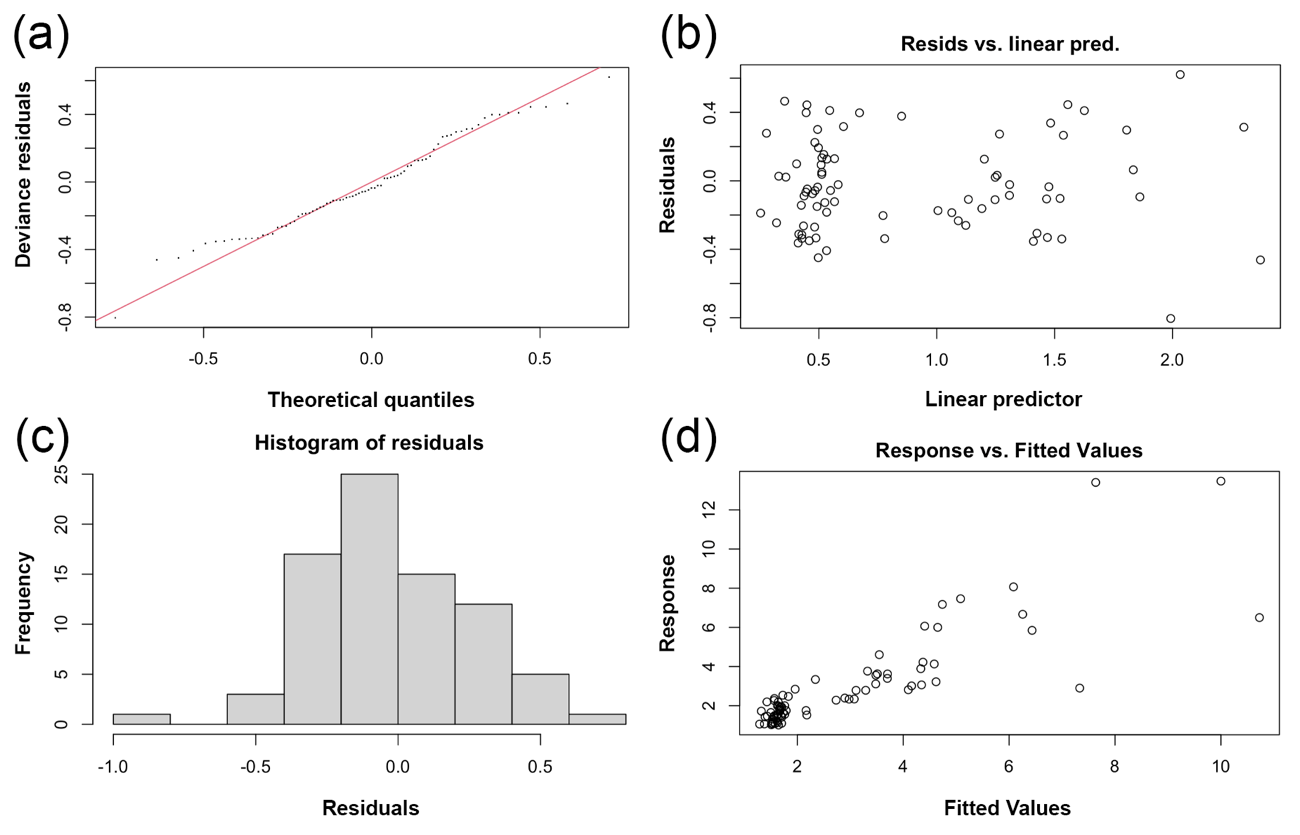
**

Fig. S5 Generalized additive model (GAM) validation of odor release from the inflorescences of *Phytelephas aequatorialis* along a day. (a) The fit of residuals to theoretical values, (b) dispersion of residues, (c) normality of residues, and (d) fitted values. Outputs were obtained using the gam.*check* function from the “mgcv” package (Wood 2011)

**REFERENCES**

[1] Wood SN. Fast Stable Restricted Maximum Likelihood and Marginal Likelihood Estimation of Semiparametric Generalized Linear Models. J R Stat Soc B. 2011; 73:3–36.

**Online Resource 3 - Insect activity**


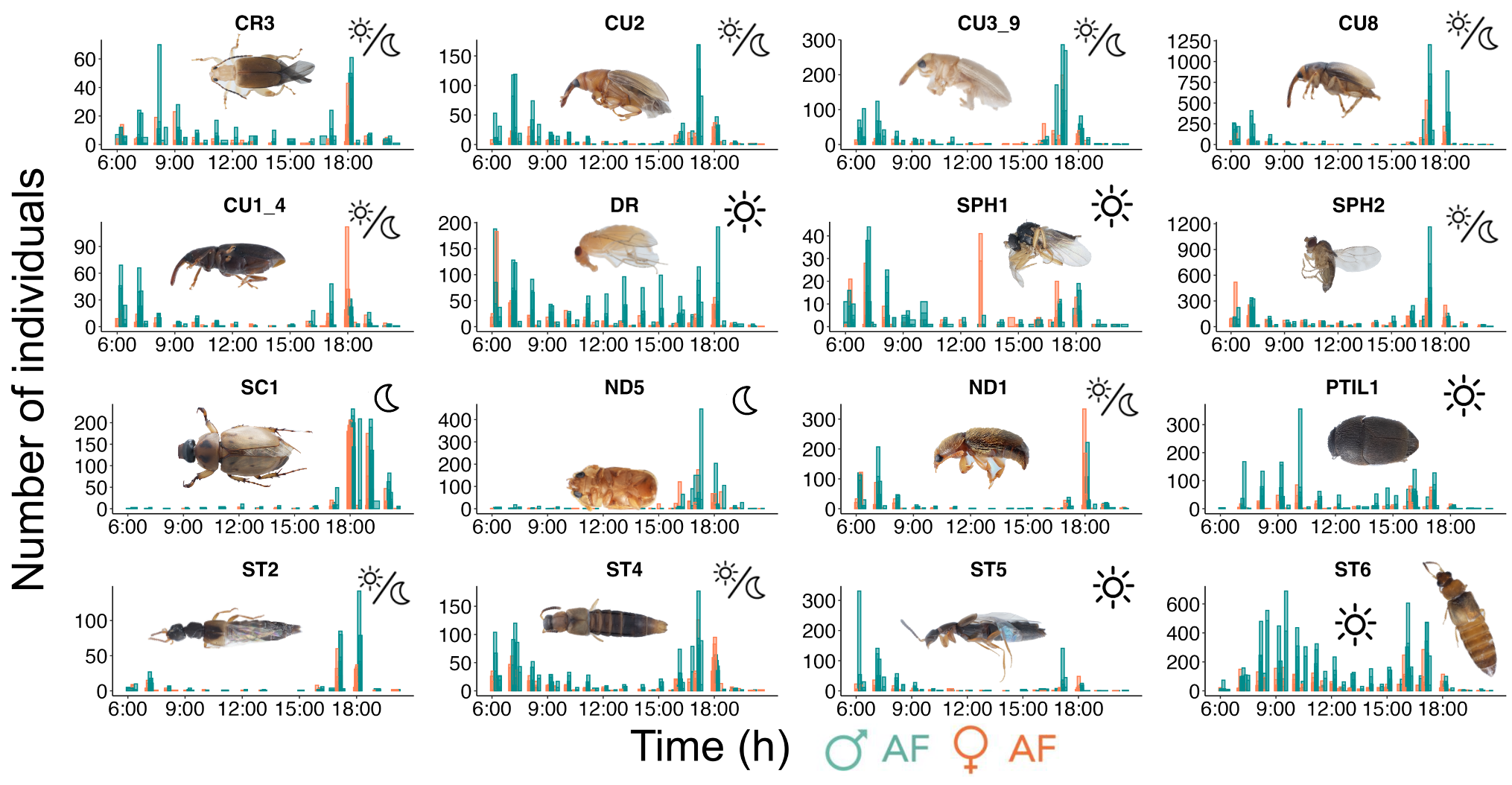


Fig. S6 Activity of insects collected per hour using artificial flowers of *Phytelephas aequatorialis* throughout the day. The orange bars represent the bioassays using female AF (time series = 12) and the blue bars the bioassays using male AF (time series = 12). The 16 selected morphospecies represent 90% of the total abundance of insects captured for female AF and male AF. The symbols in the upper right corner of each chart correspond to the habit, sun = diurnal, moon = nocturnal, sun/moon = vespertine. The bar charts are arranged according to taxonomic groups. The name of each morphospecies corresponds to the taxonomic family to which they belong: Coleoptera: CR = Chrysomelidae, CU = Curculionidae, ND = Nitidulidae, SC = Scarabaeidae, ST = Staphylinidae, PTIL = Ptilidae, Diptera: DR = Drosophilidae, SPH = Sphaeroceridae. The numbers after the taxonomy abbreviations generate a unique code in the order in which we define the different morphospecies found for each family.

In SPH1, despite the higher amounts found in female AF at the end of the day, there are no significant differences in the amount of SPH1 found in female and male AF throughout the day (Kruskal *P* = 0.15).


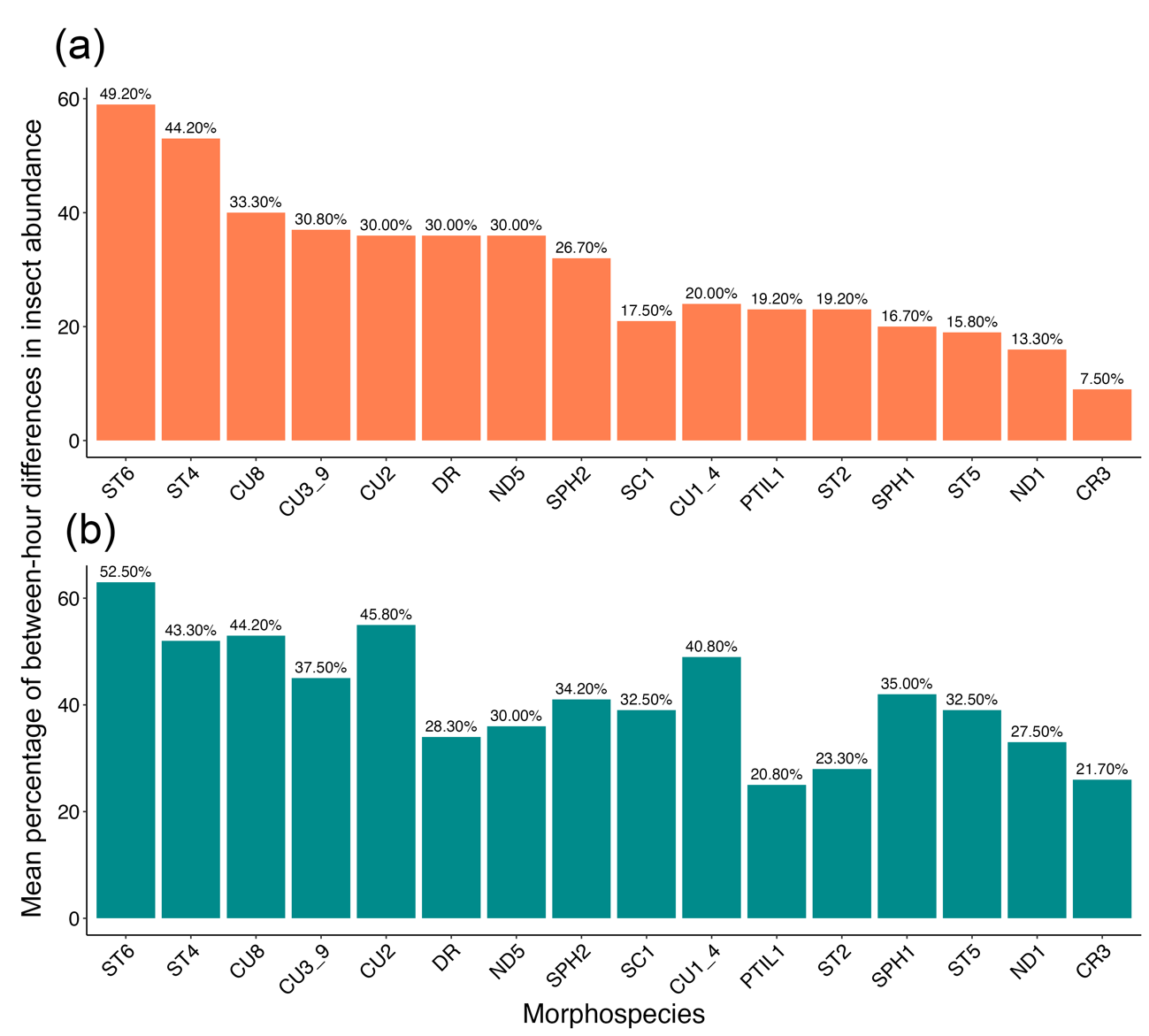


Fig. S7 Percentage of Kruskal-Wallis non-parametric tests concluding that there are significant differences between the mean number of insects captured in time intervals from all the possible combinations of time intervals with artificial flowers of *Phytelephas aequatorialis*. The orange bars represent the bioassays using female AF (time series = 12) and the blue bars the bioassays using male AF (time series = 12). The 16 selected morphospecies represent 90% of the total abundance of insects captured for female and male AF. The name of each morphospecies corresponds to the taxonomic family to which they belong: Coleoptera: CR = Chrysomelidae, CU = Curculionidae, ND = Nitidulidae, SC = Scarabaeidae, ST = Staphylinidae, PTIL = Ptilidae, Diptera: DR = Drosophilidae, SPH = Sphaeroceridae. The numbers after the taxonomy abbreviations generate a unique code in the order in which we define the different morphospecies found for each family.


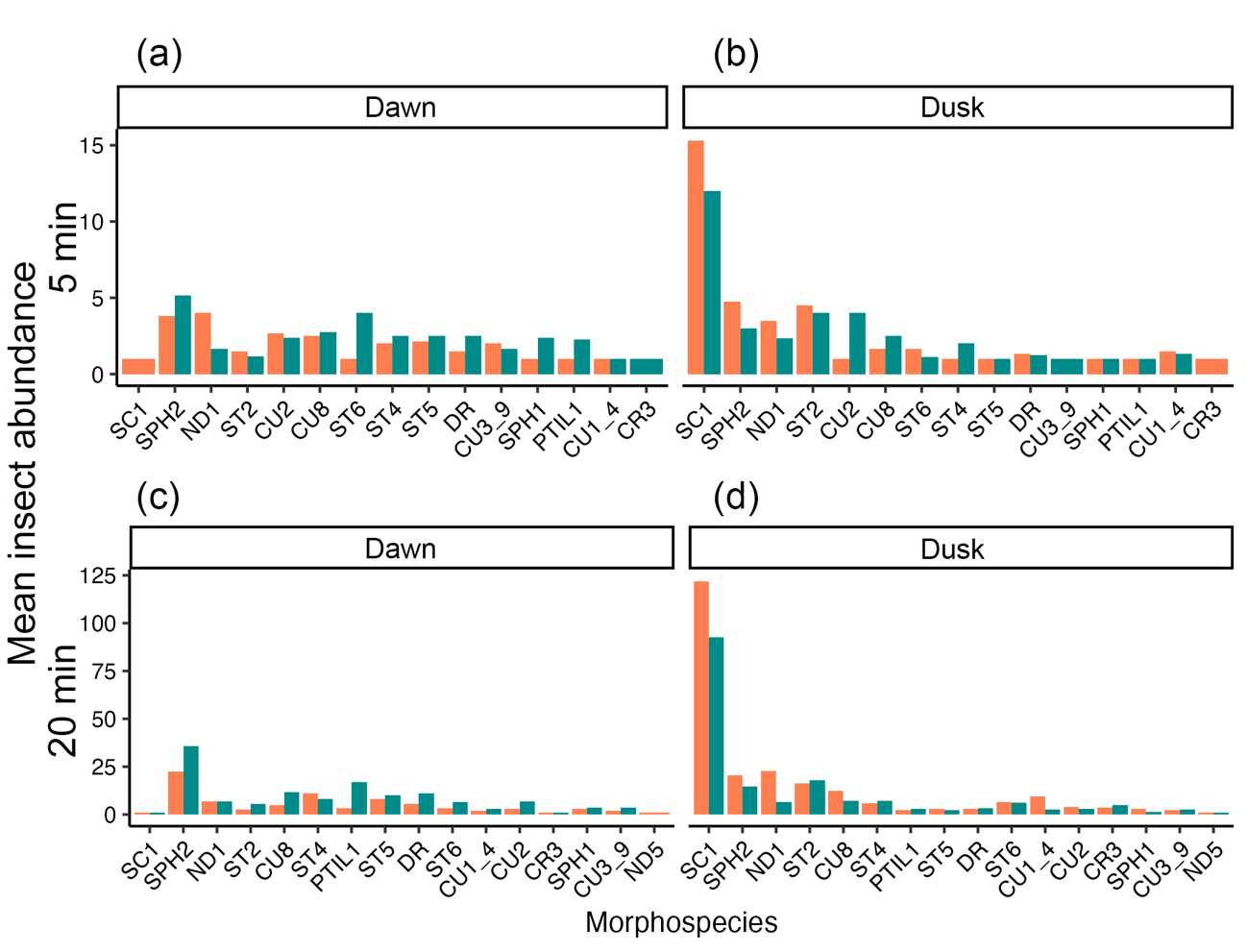


**Fig. S8 Mean number of insects caught at five minutes in the (a) dawn and (b) dusk and mean of the cumulative quantity of insects caught at 20 minutes in the (c) dawn and (d) dusk in bioassays with artificial flowers of *Phytelephas aequatorialis*.** The orange bars represent the bioassays using female AF (time series = 6) and the blue bars the bioassays using male AF (time series = 6). The 16 selected morphospecies represent 90% of the total abundance of insects captured for female AF and male AF. The name of each morphospecies corresponds to the taxonomic family to which they belong: Coleoptera: CR = Chrysomelidae, CU = Curculionidae, ND = Nitidulidae, SC = Scarabaeidae, ST = Staphylinidae, PTIL = Ptilidae, Diptera: DR = Drosophilidae, SPH = Sphaeroceridae. The numbers after the taxonomy abbreviations generate a unique code in the order in which we define the different morphospecies found for each family.

We report that in the early morning hours (dawn) the morphospecies arriving at five minutes to female AFs odor sources were ND1, SPH2, CU2, CU8, and ST5 (56% of total captures), while for male AFs the arrivals were SPH2, ST6, CU8, DR, and ST4 (51% of total captures). At dusk, the morphospecies arriving at five minutes to female AFs odor sources were SC1, and SPH2 (50% of total captures), while for male AFs were SC1, CU2, and ST2 (53% of total captures). At the conclusion of the 20-minute experiments, the dawn period revealed a greater abundance of the morphospecies SPH2, ST4, and ST5 (51% of total captures) for female AF. In contrast, for male AF, the most abundant morphospecies were SPH2, PTIL1, and CU8 (49% of total captures). At dusk, SC1 was the most abundant morphospecies for female AF and male AF (51% and 52% of total captures, respectively).

**Online Resource 4 – Floral stages**

**Table 1. Floral opening phases of one male and one female inflorescence of *Phytelephas aequatorialis* with images taken from timelapse videos.**

|  | **Bract cracking** | **Deployment start** | **Fully deployed** |
| --- | --- | --- | --- |
| **Male inflorescence** | 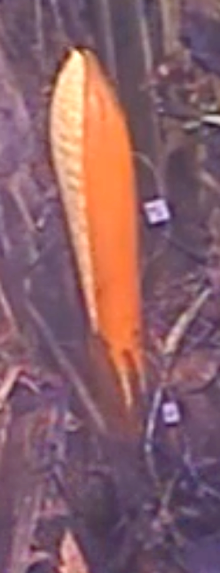 | 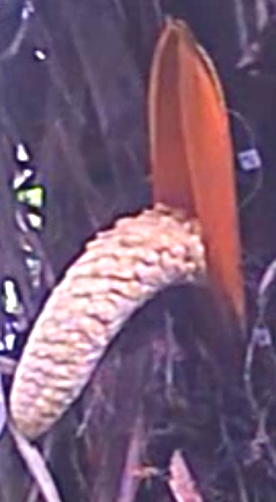 | 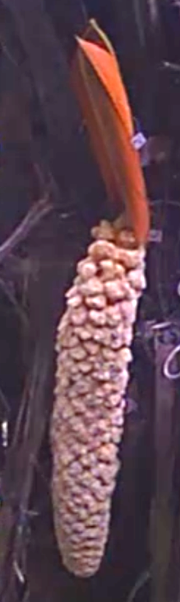 |
| **Female inflorescence** | 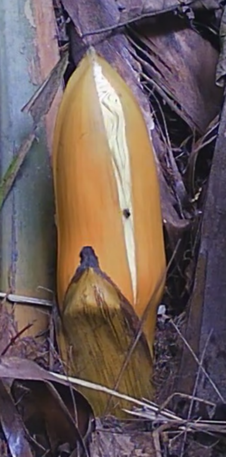 | 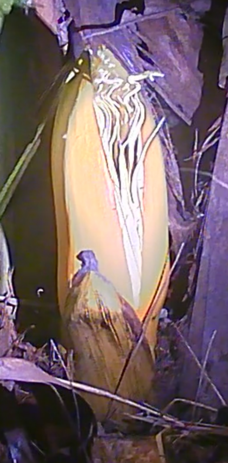 | 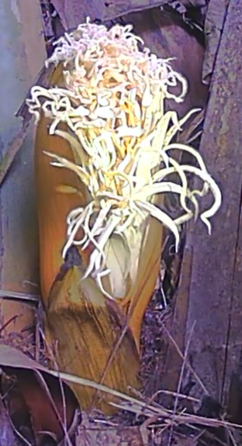 |
